# Advanced Molecular Tweezers Effectively Target Membranes Lacking Choline Headgroups for Broad-Spectrum Antiviral Efficacy

**DOI:** 10.1021/jacs.5c19450

**Published:** 2026-01-19

**Authors:** Tatjana Weil, Jan Lawrenz, Estelle Taghuo Kaptouom, Joel Mieres-Perez, Victoria Hunszinger, Konstantin M. J. Sparrer, Yasser Almeida-Hernandez, Thomas Schrader, Elsa Sanchez-Garcia, Jan Münch

**Affiliations:** † Institute of Molecular Virology, Ulm University Medical Center, 89081 Ulm, Germany; ‡ Faculty of Chemistry, University of Duisburg-Essen, 45117 Essen, Germany; § Chair of Computational Bioengineering, Department of Biochemical and Chemical Engineering, 203418 TU Dortmund, 44227 Dortmund, Germany; ∥ German Center for Neurodegenerative Diseases (DZNE), 89081 Ulm, Germany

## Abstract

Broad-spectrum antivirals are urgently required to counter present and emerging viral threats. It has previously been shown that the parental molecular tweezers CLR01 and CLR05 disrupt viral envelopes by complexing choline headgroups and that ester-functionalized “advanced” tweezers display markedly enhanced antiviral potency. Here, we determine the molecular basis of this improved activity. Using liposome leakage assays, giant unilamellar vesicles, NMR, Langmuir film balance experiments, and atomistic simulations, we demonstrate that advanced tweezers not only encapsulate choline-containing lipids but also engage lipids lacking choline headgroups via transient and conserved hydrophobic insertion events. These interactions preferentially destabilize membranes enriched in sphingomyelin, unsaturated acyl chains, or inverted cone-shaped lipids and are especially effective against small, highly curved particles resembling viral particles, explaining their broad antiviral activity for enveloped viruses. Our findings reveal a dual mechanism of action, choline binding and hydrophobic insertion, that underpins the broad-spectrum antiviral activity of advanced molecular tweezers and establish them as a promising new class of membrane-targeting antivirals for prophylactic and therapeutic use.

## Graphical abstract

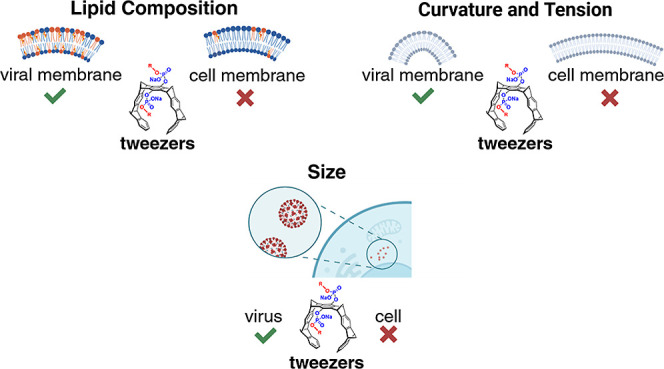

## Introduction

The emergence of the severe acute respiratory syndrome corona virus 2 (SARS-CoV-2) causing the COVID-19 pandemic^1^ along with recent outbreaks of other viruses such as monkeypox virus (MPXV)^2^ and flaviviruses, including dengue virus (DENV), yellow fever virus (YFV), and Zika virus (ZIKV),^3^ exemplify the continual viral threat to the global population. Consequently, there is a pressing need for adaptable and broad-spectrum prophylactic or therapeutic treatments.^4,5^ One promising approach for broadly active antivirals is to target properties common to multiple viruses, for example the viral membrane.^6^ During viral egress, viruses bud from the infected host cell to acquire their envelope.^7^ The viral envelope, embedded with viral glycoproteins, is crucial for infectivity and, consequently, compounds that disrupt the viral membrane display broad-spectrum antiviral activity.^8^

One class of membrane-targeting antivirals are molecular tweezers (MTs).^9,10^ MTs are supramolecular molecules that have an open cavity, which includes specific guest molecules.^11,12^ The prototype molecular tweezer, CLR01, is characterized by an electron-dense, toroidal cavity, equipped with a pair of phosphate groups at the central bridge (Figure 1a).^13^ CLR01 (Figure 1a) was originally developed as an antiamyloid agent for remodeling pathological protein aggregates^14^ and was later discovered to exhibit potent antiviral activity.^9^ Together with its analogue, the carboxylate tweezer CLR05 (Figure 1a), CLR01 has been used to study the molecular basis of antiviral activity. These parental tweezers interact with viral membrane lipids, in particular by encapsulating choline headgroups within their cavity. This binding alters lipid orientation and facilitates tweezer insertion into the bilayer, thereby increasing surface tension and ultimately disrupting the viral envelope, which results in loss of infectivity.^15–17^

**Figure 1:**
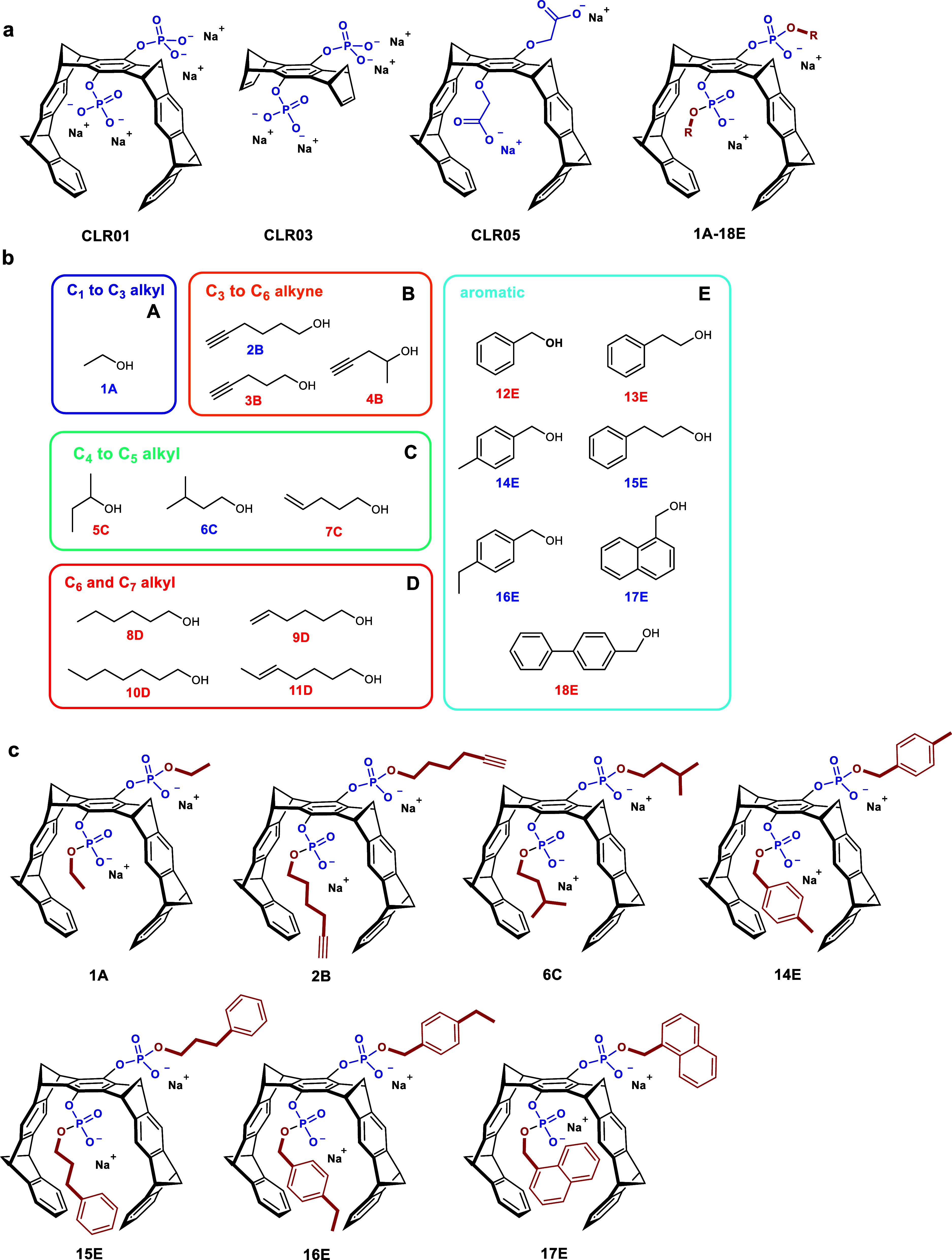
Chemical structure of tweezers. (a) Chemical structure of hydrogen phosphate tweezer CLR01, truncated tweezer CLR03, and methylene carboxylate tweezer CLR05. Right: general structure of advanced tweezers with additional ester groups 1A-18E. (b) Overview of all ester alcohols attached to the parental tweezer CLR01 for this investigation, categorized according to their size and chemical nature. (c) Chemical structure of the tweezers emphasized in this study. Adapted from Weil et al. 2022 ^18^ https://pubs.acs.org/doi/10.1021/jacsau.2c00220 with permission from the American Chemical Society. Further permissions related to this material should be directed to the ACS.

Previously, efforts to improve the antiviral efficacy of MTs led to the introduction of aliphatic and aromatic ester groups into the side arms of CLR01 (Figure 1b).^18^ Subsequent structure–activity relationship analyses identified derivatives with C6/C7 alkyl and aromatic tweezers as the most potent, exhibiting enhanced antiviral activity and high selectivity indices (SI), indicative of substantial therapeutic potential. Biomolecular modeling showed that these advanced tweezers retain the ability to bind the choline head groups of phosphatidylcholine (PC) and sphingomyelin (SM), altering lipid orientation, as observed with CLR01. Moreover, these advanced MTs demonstrated the capability to integrate their side arms into the viral membrane, which correlates with an elevated tension and a heightened disruption efficacy. Advanced MTs were shown to inhibit a wide range of enveloped viruses, including SARS-CoV-2, human immunodeficiency virus type 1 (HIV-1), measles virus (MeV), influenza A virus (IAV), and respiratory syncytial virus (RSV).^18^ Lead MTs with the highest antiviral efficacy and lowest cytotoxicity are currently being explored in advanced preclinical development stages to be administered as sprays or aerosols to combat respiratory virus infections.

Importantly, the interactions of MTs with lipid constituents other than PC and SM of viral and cellular membranes, as well as the specificity of advanced tweezers for these lipids, remain unexplored. Cellular membranes are intricately assembled with a heterogeneous mix of lipids, primarily phospholipids, sphingolipids, and sterols, which vary in composition, shape, tail length, saturation, and charge, influencing membrane characteristics.^19,20^ While the detailed architecture of viral membranes is less understood, the cellular membranes of the endoplasmic reticulum (ER), Golgi, endoplasmic reticulum-Golgi intermediate compartment (ERGIC), and plasma membrane (PM), all serve as viral budding sites and are well-characterized. The ER and Golgi are predominantly composed of phospholipids, especially phosphatidylcholine (PC) and phosphatidylethanolamine (PE),^21^ with smaller amounts of other phospholipids such as phosphatidylserine (PS), phosphatidylinositol (PI), phosphatidic acid (PA), and phosphatidylglycerol (PG).^20,22^ These membranes are also rich in unsaturated fatty acid chains.^20^ Ceramide, the precursor of sphingolipids, along with cholesterol, is synthesized in the ER,^20^ followed by the conversion of ceramide into various sphingolipids within the Golgi; however, both organelles contain only trace amounts of sterols and sphingolipids. By contrast, the PM is considerably enriched with sphingolipids and sterols, particularly within lipid rafts.^23,24^

Here, we determined the lipid selectivity of molecular tweezers with liposomes comprising the most prevalent lipids identified in cellular and viral membranes.^20^ When these liposomes were exposed to selected MTs, we observed that most of the advanced tweezers lysed a wider array of liposome types with increased efficacy compared with their predecessors, CLR01 and CLR05. Notably, all tweezers exhibited an augmented lysis sensitivity toward membranes enriched with sphingomyelin (SM), those with a higher saturation degree, or lipids with an inverted cone shape, which contribute to elevated membrane fluidity and permeability. Biomolecular modeling of these advanced tweezers interacting with diverse lipid types revealed a propensity for hydrophobic contacts with the liposomal membranes. This marked a departure from the previously understood mechanism where the formation of inclusion complexes with lipids, such as PC and SM, was the driving factor for the tweezer’s effect. Extending these insights, we evaluated the efficacy of tweezers against viruses originating from different budding sites, which affect the lipid composition and properties of the viral membrane. Our investigation covered viruses budding from the PM,^25–27^ ERGIC,^28–30^ ER,^31,32^ and Golgi apparatus^33^ and revealed that tweezer activity correlates with the lipid composition of viruses from identical budding sites. Moreover, the tweezers demonstrated selectivity for smaller, highly curved particles, akin to the viral envelope. Finally, we expanded upon previous studies demonstrating that molecular tweezers (MTs) inhibit SARS-CoV-2, Influenza, and RSV by showing that MTs are also effective against common cold coronaviruses and herpes simplex viruses (HSV-1 and HSV-2). Given that MTs are developed for inhalative or topical applications, their broad-spectrum activity against these respiratory viruses and herpesviruses highlights their potential for use in localized treatments. This supports their continued development as targeted antiviral agents suitable for both inhalation and topical administration.

## Results

### Advanced Tweezers Have a Broad Membranolytic Activity on Various Lipid Types

The antiviral properties of advanced molecular tweezers (Figure 1) have been previously described,^18^ but their lipid specificity has not been thoroughly explored. To investigate the specificity of advanced tweezers for viral membrane lipids, we produced a set of 10 liposome types containing the most prevalent phospholipids, sphingolipids, and sterols found in membranes (Figure 2a,b). Due to the inability of some lipids to form stable liposomes independently, the most common lipid PC was combined with these lipids at a 50/50 ratio.^21^ A 100 nm pore size membrane was chosen to produce a uniform size of liposomes (resembling the size of many enveloped viruses), which were filled with the self-quenching dye carboxyfluorescein (Figures 2b and S1).^15^ The zeta potential of these liposomes varied, with PC liposomes at 1.03 mV and PG liposomes at −39.65 mV, reflecting the physical properties of the lipids, where PC and PE are neutral and PS, PG, PA, and PI are negatively charged (Figure 2b).^34^ The presence of cholesterol (Chol) or SM in the membranes was found to lower the zeta potential, confirming prior analyses.^35,36^

**Figure 2:**
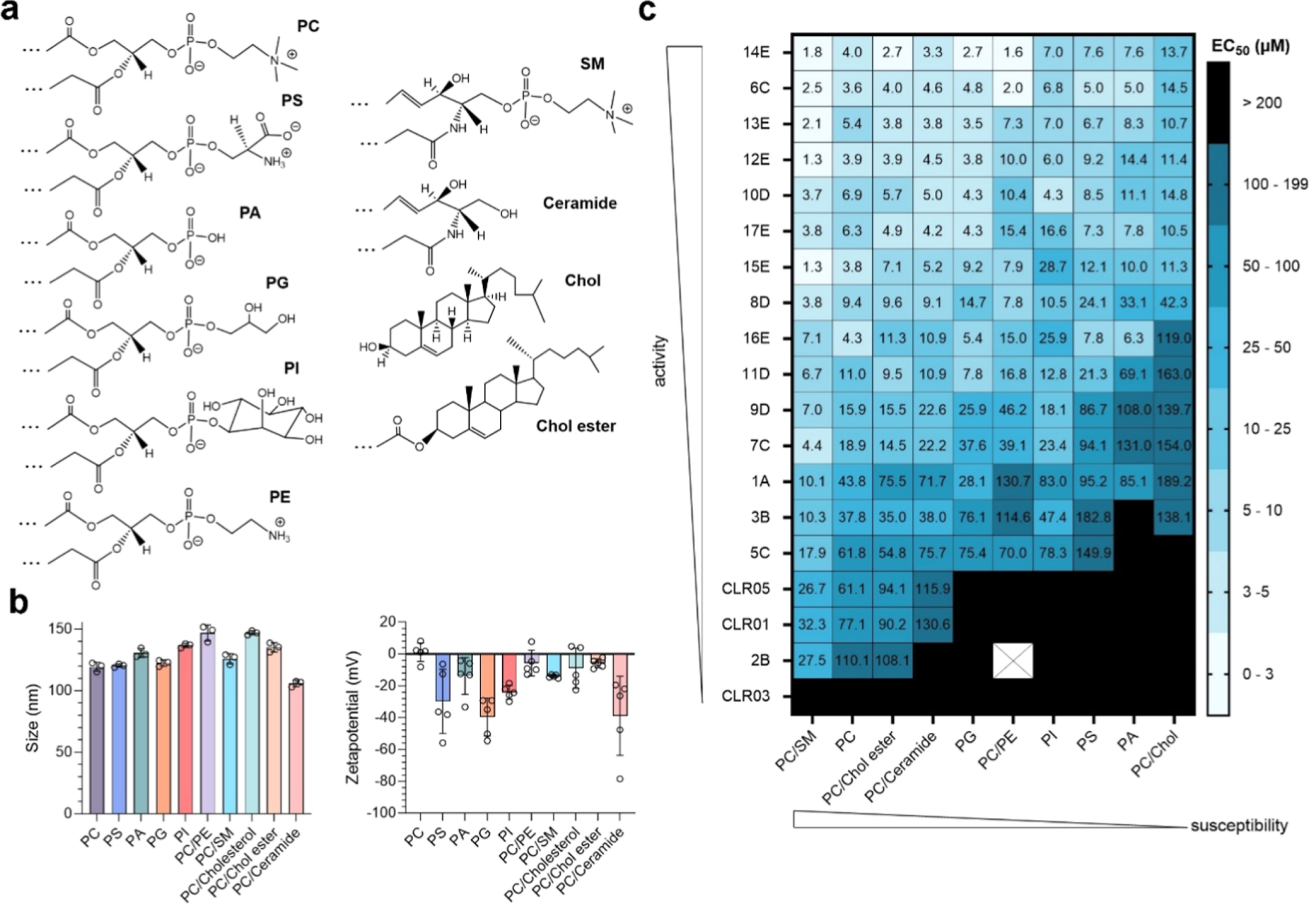
Advanced MTs demonstrate a broad membranolytic capacity across various lipid compositions. (a) Chemical structure of lipid head groups; generated with ChemDraw. (b) Particle size and zeta potential of 100 nm sized uniform or mixed (50/50 mol %) liposomes, filled with 50 mM carboxyfluorescein and analyzed by NTA. Measurement was conducted in 3 (NTA) to 5 (zeta potential) acquisitions and shown as mean values ±SD (raw data are shown in Figure S1). (c) Liposomes (2.5 × 10^10^ particles/mL) were incubated with increasing concentrations of tweezers for 30 min, and fluorescence was recorded every minute at 485 nm excitation and 528 nm emission. Baseline was measured for 5 min in the absence of tweezers, and the maximum fluorescence was recorded after the addition of 1% Triton X-100. Values were corrected for baseline, normalized to maximum fluorescence, and area under to curve was blotted (shown in Figure S2). Graphs show overview of calculated EC_50_ derived from Figure S2. Modified from Weil, 2023 ^37^ (CC BY 4.0; https://creativecommons.org/licenses/by/4.0/).

The MTs were assessed for their ability to disrupt the prepared liposomes in a dye leakage assay. All advanced MTs, except for the alkyne tweezer 2B, displayed enhanced membranolytic activity across different liposome types as compared to their predecessors, CLR01 and CLR05, indicating a broader and more effective disruption capability (Figures 2c and S2). Prior research indicated that the inclusion of lipid head groups into the cavity of CLR01 and CLR05 is exclusive to those with a choline headgroup.^15^ This binding mode of CLR01 and CLR05 was corroborated by the current testing against a variety of liposome types, whereas the negative control tweezers, CLR03, showed no activity. However, advanced MTs disrupted all liposomes, and performed best on PC/SM liposomes (EC_50_: 1.3 μM in case of 15E to 32.3 μM for CLR01), while disruption of PC/Chol required the highest tweezer concentrations (EC_50_: 10.5 μM in case of 17E to 189.2 μM for 1A) (Figures 2c and S2). Improved activity of all tweezers in the presence of SM confirms previous studies showing higher binding affinities of CLR01 toward sphingomyelin.^15^ Interestingly, the presence of SM increased the lytic activity of the tweezer in all cases, except for 16E, which however showed the highest activity against phospholipid liposomes. The tweezer activity against PC/Chol ester or PC/ceramide liposomes was slightly lower than against uniform PC liposomes, but increased compared to phospholipid PG, PE, PI, PS, and PA liposomes (Figure 2c). The aromatic tweezer 14E emerged as the most effective, although other aromatic and C6/C7 alkyl tweezers also showed increased membranolytic activity. In summary, the advanced molecular tweezers demonstrated a significantly broadened lipid-binding spectrum compared to that of CLR01 and CLR05, showcasing enhanced membranolytic activity across various liposome types. This indicates that interactions beyond those with choline head groups are responsible for the increased efficacy of these advanced molecular tweezers.

### NMR Titrations and Film Balance Experiments of MTs and Lipids

To study the direct interaction between advanced MTs and lipids, we performed NMR titrations of the tweezer derivatives with lipid molecules, relying on significant upfield shifts indicative of a cation’s inclusion in the tweezer cavity. This method allows us to discern interactions specifically with lipids that have a choline headgroup, as lipids lacking this feature do not enter the tweezer cavity and thus do not produce noticeable signal shifts. We focused on the direct interactions with the lipids 1,2-dioleoyl-*sn*-glycero-3-phospho-choline (DOPC) and sphingomyelin (SM) under identical experimental conditions. The results showed that the sensor protons within the trimethylammonium headgroup of the choline experienced substantial upfield shifts when included inside the tweezer cavity, confirming their interaction (Figure S3). The dissociation constants (*K*_d_) for these 1:1 complexes were found to be in the millimolar range, similar to those observed for CLR01, indicating that the tweezers–lipid interactions are of comparable strength. The nature of the tweezer’s ester arm was found to modulate the affinity of these interactions. Specifically, in DOPC, tweezer derivatives with hexyl arms formed more stable complexes with the lipids compared to those with hexynyl arms, likely due to electrostatic repulsion between the π-electrons of the hexynyl’s triple bond and the lipid’s phosphate group.

Conversely, tweezers with aromatic arms, particularly those without the ethylbenzyl group, showed enhanced affinity, highlighting how even minor structural differences can significantly influence the lipid interaction. Moreover, when testing the lipid raft-forming phosphosphingomyelin SM, the affinities for the previously problematic hexynyl and ethylbenzyl tweezers improved, although the affinities for other tweezers remained unchanged (Figure S3). This suggests that the direct affinity between the tweezers and lipid molecules does not differ significantly between DOPC and SM. However, it is clear that aromatic tweezers generally exhibit superior affinity compared to aliphatic ones, which correlates with their lower IC_50_ values for viral inhibition in cell culture, possibly due to additional π-cation interactions. Thus, the attractive interactions between the molecular tweezers and encapsulated lipid guests, particularly when adjacent lipid molecules in the membrane are identical, can lead to substantial changes in membrane destabilization. Surprisingly, despite similar lipid affinities between advanced tweezers and CLR01, their antiviral activities do not directly correlate, suggesting that factors beyond mere lipid affinity, such as the insertion of the tweezers’ side arms into the viral membrane, may play critical roles in enhancing antiviral efficiency, a phenomenon that cannot be fully assessed by NMR measurements alone.

To examine the impact of MT interactions with lipid membranes on the surface tension, film balance experiments were performed. Our experiments were limited to choline-containing lipids since no inclusion event occurs with neutral lipids, thereby defining the experimental parameters. Pure DOPC and SM were spread as a monolayer at the air–water interface, and molecular tweezers were subinjected into the aqueous phase. In all cases a substantial increase of the area under the curve (p/A) indicated incorporation of the tweezers into the lipid monolayer (Figure S4) in line with the proposed mechanism. When compression of the tweezer-containing monolayer started, the p/A curves became considerably steeper, providing experimental evidence of elevated surface pressure. In our model, this originates from the tight inclusion of the choline head into the tweezer cavity with a tilted orientation. At a certain pressure, the p/A curve leaned back and became horizontal, a typical behavior when the lipid layer is disrupted. Advanced tweezers 8D and 14E produced stronger effects than ancestor CLR01. We conclude that massive changes in p/A diagrams reflect major consequences inside the viral membranes, strongly supporting the elevated surface tension after tweezer docking and demonstrating the resulting destabilization of the doped membrane.

### Binding of Advanced Tweezers to Phospholipids Lacking the Choline Headgroup Is Based on Hydrophobic Interactions

To explore how advanced tweezers affect phospholipids without the choline headgroup, biomolecular modeling studies were conducted with 14E and 6C, the most membranolytic tweezers (Figure 2). The aromatic tweezer 14E exhibits a higher activity in the experiments with the pure 16:0–18:1 PG/1-palmitoyl-2-oleoyl-*sn*-glycero-3-phospho-(1′-rac-glycerol) (POPG) membrane (Figure 2c). Extensive molecular dynamics (MD) simulations (see Supporting Information) of 14E and a POPG model membrane showed mostly transient binding and unbinding events of the tweezer with the membrane and only one instance of conserved binding (Table 1, Figures 3a and S6). This behavior is different from that observed for 14E with the DOPC/SM/Chol model membrane previously studied by us.^18^ In the case of 6C with POPG, only one conserved binding event was found, but fewer transient binding events took place with respect to the 14E/POPG system (Figure S7). Unlike DOPC and SM, POPG does not feature a positively charged headgroup, just two hydroxyl groups. Consequently, we found that for all the modeled lipid bilayers (POPG, 16:0–18:1 PS/1-palmitoyl-2-oleoyl-*sn*-glycero-3-phospho- *l* -serine (POPS) and 16:0–18:1 PA/1-palmitoyl-2-oleoyl-*sn*-glycero-3-phosphate (POPA)), no inclusion complexes are formed. Instead, our simulations indicate a mechanism where hydrophobic interactions between the tweezers and lipids may lead to membrane destabilization.

**Table 1:** Total Number of Conserved and Transient Binding Events of the Tweezers in the Model Lipid Bilayers

tweezer—lipid	conserved binding events	transient binding events
6C—POPG	1	5
14E—POPG	1	28
6C—POPA	4	9
14E—POPA	3	5
6C—POPS	6	1
14E—POPS	8	2

**Figure 3:**
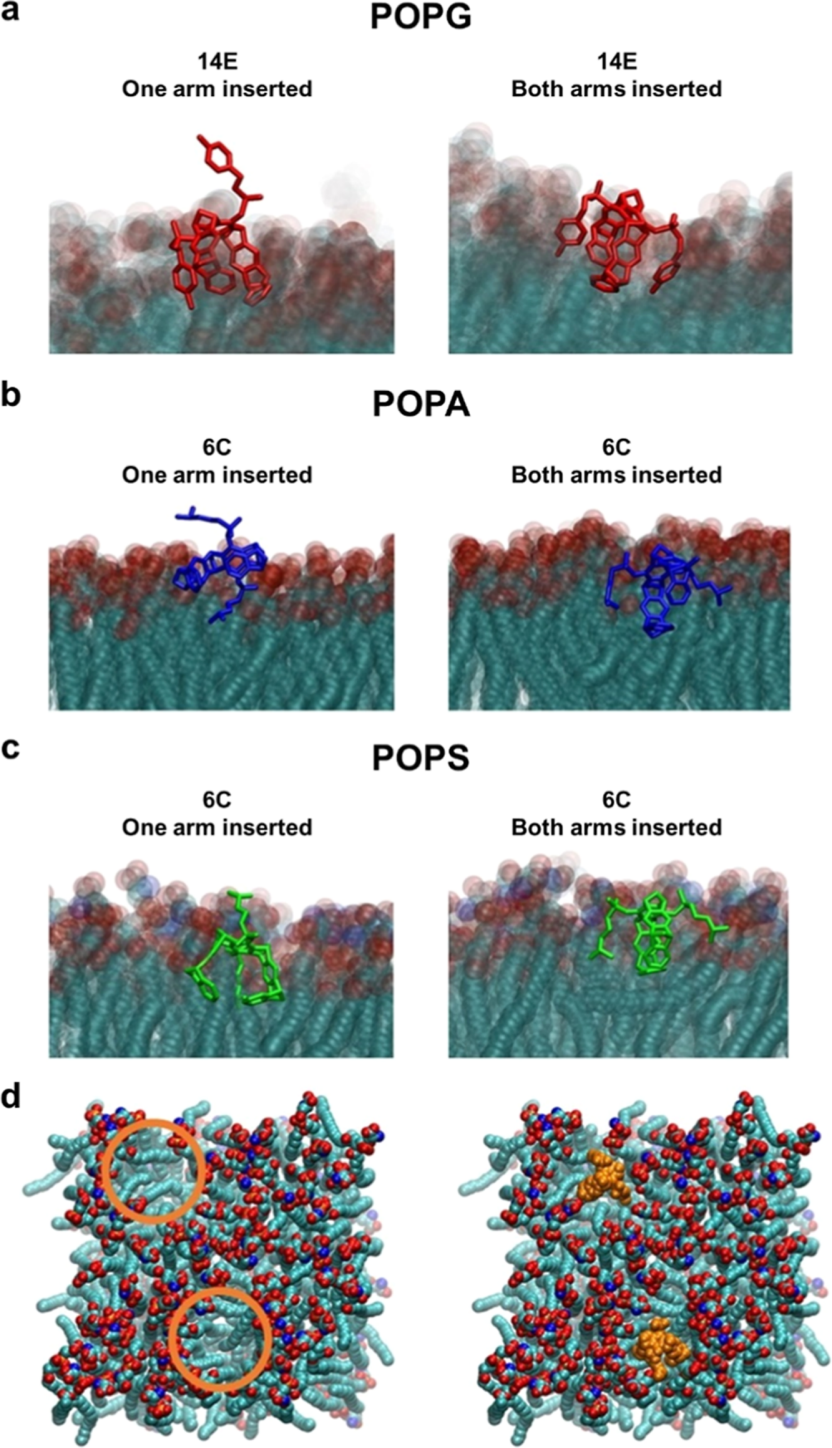
Representative interactions of advanced tweezers with phospholipids in the bilayers. (a) The molecular tweezers (14E shown as an example, in red sticks) can insert one or two side arms into the POPG membrane. During most of the MD simulations, the insertion occurs via transient binding and unbinding events. (b,c) Both tweezers (6C shown as an example, in blue or green) can insert one or two side arms into POPA and POPS bilayers. The membranes are rendered in transparent representation to allow the visualization of the tweezer arms. (d) Both tweezers (6C shown in orange, right) bind into hydrophobic patches (orange circles, left) of the POPS membrane. Water molecules, ions, and hydrogen atoms are omitted for clarity.

Simulations revealed hydrophobic patches in POPA and POPS membranes, as a result of the exposure of the acyl chains of these phospholipids, in agreement with a previous study of PA-containing membranes (Figure 3d).^38^ The simulations show how the tweezers interact with these patches via a novel mechanism (Figure 3b). The tweezer can insert its hydrophobic core and arms into the POPA and POPS membranes (Figure 3b,c) thus establishing conserved binding events with the membranes (Table 1, Figures S8–S11). Our simulations suggest that the binding of the advanced tweezers may stabilize the hydrophobic patches of the membrane, which could lead to subsequent destabilization of the bilayer (Figure 3d). This effect can be compared to a “lawn mower” where the tweezer interacts with the membrane patches and diffuses laterally embedded in the lipid leaflet. 6C and 14E adopt several diverse orientations with respect to the membrane during the conserved binding events (Figures S6B–S11B).

### Tweezer Membranolytic Efficiency Depends on the Shape and Saturation of the Lipid

Disrupting PC/Chol and PA liposomes required the highest concentrations of tweezers, with not all achieving complete leakage (Figures 2c and S2). A possible explanation lies in the molecular shape of the lipids: PA and Chol, along with PE, have small head groups, creating a cone shape that leads to negative curvature and membrane compression (Figure S12a).^19,39,40^ Conversely, lyso-lipids with a single fatty acid tail have an inverted cone shape, which increases membrane permeability, stress, and tension.^41^ These lipids are found in mammalian cell membranes with concentrations under 5%. In addition, the presence of cholesterol likely increases membrane order and stiffness, thereby reducing lipid mobility and hindering tweezer insertion into the bilayer interior.^42^ As lyso-lipids tend to form micelles at higher concentration,^41^ we produced lyso-lipid/PC liposomes at a 5/95 mol % ratio showing uniform particle size and varied zeta potentials (Figure S12b–d). Lyso-PS liposomes had the most negative zeta potential (−42.64 mV), with lyso-lipids generally lowering the zeta potential. With the exception of CLR03, tweezers disrupted lyso-lipid liposomes dose-dependently (Figure S12e) showing elevated activity compared to PC liposomes, with EC_50_ values from 0.9 to 76.5 μM, most likely due to the shape-related permeability of lyso-lipids (Figure S12f,g).

Tweezer activity may also be affected by the saturation degree of the fatty acid tails. Saturated lipids, whose level increases along the secretory pathway and which are abundant in the PM, form stable, tightly packed liquid-ordered (Lo) domains that reinforce the PM against stress (Figure S13a).^19,43^ In contrast, unsaturated lipids, with their kinked acyl chains, form liquid-disordered (Ld) domains common in the ER and Golgi membranes, and reduce stability.^44^ Experiments were conducted with 100 nm PC liposomes with saturated and varied unsaturated fatty acid tails, showing that all tweezers disrupted unsaturated PC liposomes, while only aromatic tweezers 14E and 13E induced over 50% leakage in saturated PC 18:0 liposomes (Figure S13a–c). A high number of double bonds made liposomes more vulnerable to tweezer disruption (Figure S13d), indicating that higher fluidity of lipid tails enhances tweezer efficacy. Thus, tweezer-induced membrane disruption efficiency is influenced by lipid shape, with lyso-lipids enhancing permeability and by fatty acid saturation, with unsaturated lipids exhibiting greater vulnerability to disruption.

### Molecular Tweezers Are More Effective against Smaller Particles

To explore the correlation between tweezer activity and particle size, a key factor distinguishing viral particles from cells, we created virus-like liposomes (DOPC/SM/Chol at 45/25/30 mol %) of various sizes by extruding through membranes with 50–800 nm pores and characterized them using dynamic light scattering (DLS) (Figure 4a). Dye leakage assays with the original tweezer CLR01 and advanced candidates 10D (C7 alkyl) and 14E (aromatic) showed higher activity against smaller particles (Figure S14). Adjusted for particle numbers, tweezer activity decreased with increasing particle diameter (Figure 4b,c) with constant CLR01 concentration showing increased activity as particle count decreased (Figure S15).^16,18^

**Figure 4:**
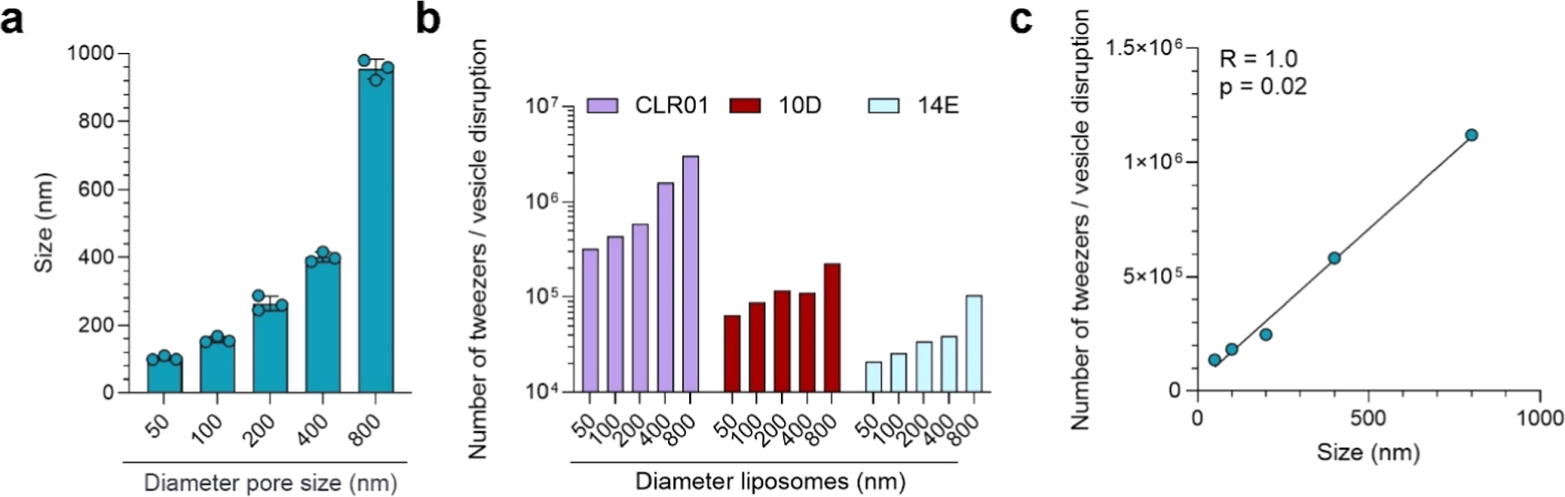
Molecular tweezers are more effective against smaller particles. (a) Virus-like liposomes composed of DOPC/SM/Chol (45/25/30 mol %) and filled with a 50 mM self-quenching dye were sized using membranes with pore diameters ranging from 50 to 800 nm and characterized by DLS. The graph displays the average *z*-size from three readings ±SD. (b) To assess tweezer efficacy, varying concentrations were incubated with a fixed number of liposomes for 30 min, with dye leakage monitored at 485 nm excitation and 528 nm emission wavelengths. Baseline fluorescence was established over 5 min without tweezers, and maximum fluorescence was determined post 1% Triton X-100 addition. Readings were baseline-corrected and normalized to the maximum fluorescence, with the area under the curve plotted and EC_50_ values calculated (refer to Figure S15). The graph illustrates normalized quantities of tweezers needed to disrupt a single particle, based on EC_50_. (c) Finally, the average number of CLR01 tweezers required to destroy one liposome was plotted against the liposomes’ measured sizes, with analysis via Spearman correlation and a two-tailed *p*-value. Modified from Weil, 2023 ^37^ (CC BY 4.0; https://creativecommons.org/licenses/by/4.0/).

We further studied molecular tweezer effectiveness against giant unilamellar vesicles (GUVs) with virus-like lipid membranes (DOPC/SM/Chol at 45/25/30 mol %) sized at 5.7 μm and filled with the self-quenching dye carboxyfluorescein (Figure S16a). Among the 19 molecular tweezers tested, only CLR03 showed no lytic activity (Figure S16b,c). EC_50_ values for inducing leakage ranged from 0.4 μM for aromatic tweezer 17E to 185.8 μM for alkyne tweezer 2B, with some tweezers not achieving complete leakage at maximum concentrations. Comparing GUV to 200 nm virus-like liposome assays showed similar EC_50_ values, indicating comparable effectiveness (Figures 5a and S16d). However, normalizing for particle count revealed tweezers were 2.2 × 10^5^ times more active on liposomes. Even when adjusted to the number of lipids per vesicle, liposomes exhibited a significant 260-fold increase in activity compared to GUVs, highlighting the impact of particle curvature on tweezer efficacy. Individual tweezer analysis showed aromatic tweezers had the lowest selectivity difference between liposomes and GUVs (26.3 to 96.4-fold), while C6 and C7 alkyl tweezers like 8D, 10D, and ancestors CLR01 and CLR05 demonstrated higher selectivity, with 2B showing the greatest liposome selectivity (613-fold), correlating with low cytotoxicity and high selectivity index (SI) values (Figure 5b).^18^ Taken together, MTs demonstrate significantly higher efficacy against smaller liposomes compared to GUVs, highlighting the crucial role of particle size and curvature in their membrane-disrupting activity, with alkyl tweezers exhibiting greater selectivity and lower cytotoxicity.

**Figure 5:**
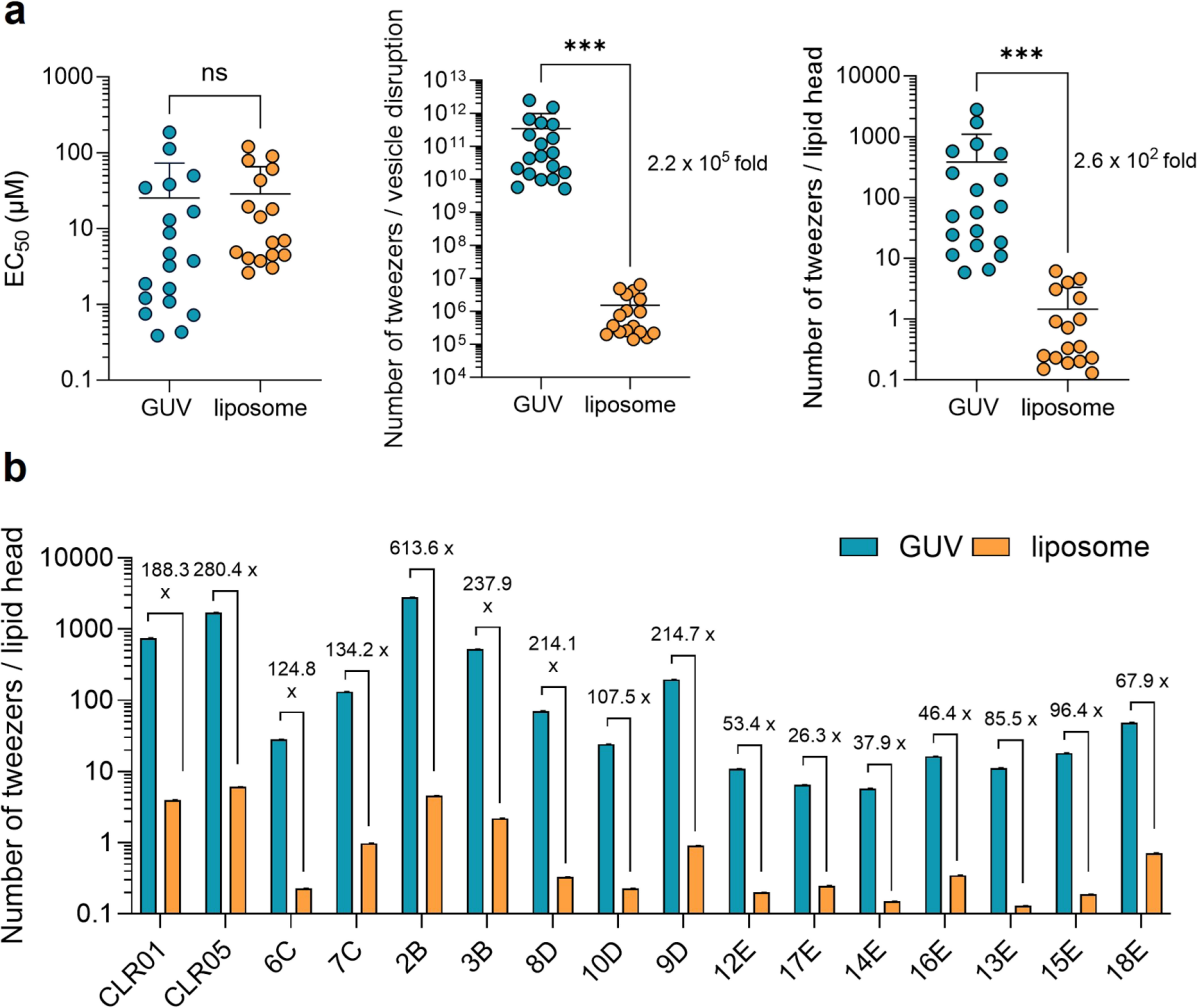
Molecular tweezers are less effective against larger giant unilamellar vesicles (GUVs). (a) GUVs, consisting of DOPC/SM/Chol (45/25/30 mol %), were filled with 50 mM carboxyfluorescein. When treated with serial dilutions of tweezers for 30 min, dye leakage was monitored at 485 nm excitation and 528 nm emission. Baselines were established without tweezers for 5 min, and maximum fluorescence was determined after adding 1% Triton X-100. Values were corrected for baseline, normalized to maximum fluorescence and area under to curve was plotted (shown in Figure S16b). The three graphs display the mean EC_50_ values, from Figure S16c and previous data;^18^ the number of tweezers needed to disrupt one GUV or liposome, and the number of tweezers per lipid head required for particle disruption. Statistical analysis was performed using the nonparametric Mann–Whitney test (*** = *p* < 0.001). (b) Individual comparison of tweezer activity against GUVs and liposomes, derived from (a). Modified from Weil, 2023 ^37^ (CC BY 4.0; https://creativecommons.org/licenses/by/4.0/).

### Broad-Spectrum Antiviral Efficacy of Molecular Tweezers against Common Cold Coronaviruses and Herpesviruses

CLR01 and CLR05 have demonstrated broad-spectrum antiviral effects against various enveloped viruses, including HIV-1, ZIKV, and HSV-1.^9,15,16^ Advanced tweezers retain this broad activity and also effectively inhibit infections by SARS-CoV-2, MeV, RSV, and HIV-1.^18^ Despite their efficacy, these tweezers are inactivated in serum due to interactions with serum components,^16,17^ highlighting the need for localized administration through inhalation or topical applications. We evaluated their effectiveness against additional respiratory viruses, the three common cold coronaviruses hCoV-NL63, -229E, and -OC43, and HSV-1 and HSV-2, which are also amenable to topical treatment. Our results indicate that advanced tweezers exhibit significantly enhanced antiviral activity against all three common cold coronaviruses and both herpesviruses, with notably lower IC_50_ values compared to those of CLR01 (Figures 6a,b and S17). The antiviral activity of these tweezers was dose-dependent across the tested viruses, demonstrating their potential as highly effective agents for localized antiviral therapies. Given the broad-spectrum activity and enhanced effectiveness of advanced tweezers, they represent promising tools for pandemic preparedness, offering a potent option for rapid response to emerging respiratory viruses and herpesvirus infections through localized treatment methods.

**Figure 6:**
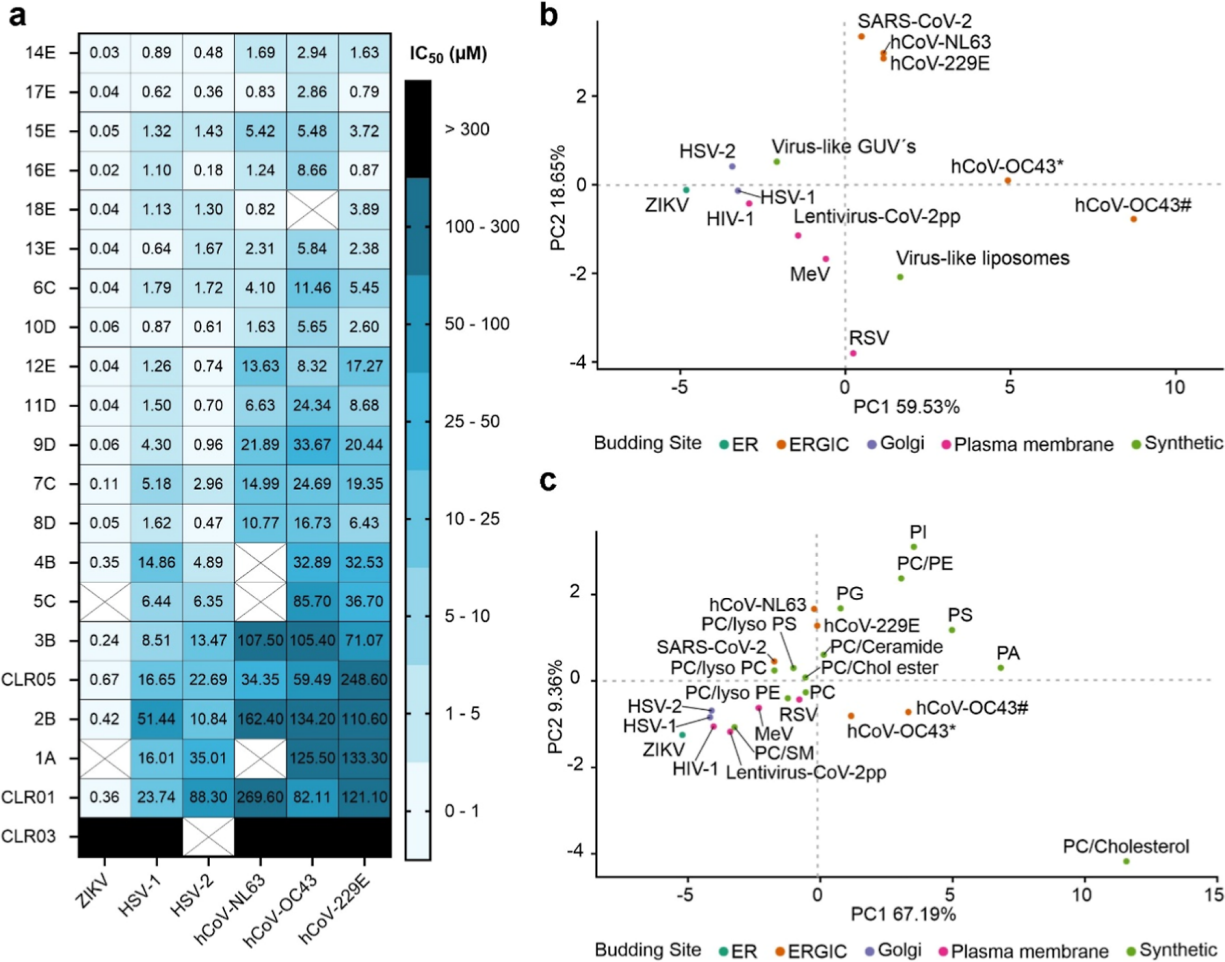
Broad-spectrum antiviral activity of advanced tweezers. (a) Human coronaviruses NL63, 229E, and OC43 were preincubated with molecular tweezers and used to infect Caco-2 or Huh-7 cells, depending on the virus strain. Viral nucleocapsid expression was quantified by in-cell ELISA. ZIKV and GFP-encoding HSV-1 and HSV-2 were treated similarly and infection of Vero or ELVIS cells was measured by in-cell ELISA (ZIKV) or β-galactosidase activity (HSV). Results represent IC_50_ values of the tweezers against the indicated viruses, calculated from the assays shown in Figures S17 and S18. (b) Principal component analysis (PCA) of IC_50_/EC_50_ values from (a), Figures S16c and S19b, and earlier work,^18^ showing patterns of tweezer activity across different viruses and virus-like particles. *hCoV-OC43 was produced in TMPRSS2-expressing Vero E6 cells; #hCoV-OC43 was produced in HCT-8 cells. (c) PCA incorporating IC_50_/EC_50_ values from (c) together with data from Figures 2c and S12f, illustrating clustering of viral activity with lipid liposome specificity. Modified from Weil, 2023 ^37^ (CC BY 4.0; https://creativecommons.org/licenses/by/4.0/).

### The Antiviral Efficacy of Molecular Tweezers Is Influenced by the Site of Viral Budding

Molecular tweezers display broad-spectrum activity against many enveloped viruses; yet, their antiviral potency differs between virus families. Here, we investigated whether these differences are related to the site of viral budding, which determines the lipid composition of the viral envelope. Viruses such as MeV, RSV, and HIV-1 bud from lipid rafts in the plasma membrane (PM)^25–27^ resulting in envelopes enriched in cholesterol (Chol) and sphingomyelin (SM).^45–47^ Consistently, tweezer activity against these viruses correlated well with PM-mimicking liposomes composed of DOPC/SM/Chol (45/25/30 mol %).^18^ In contrast, SARS-CoV-2, which buds from the ERGIC,^28^ showed different susceptibility patterns, reflecting its distinct lipid composition. Testing of additional ERGIC-budding coronaviruses^29,30^ confirmed that advanced tweezers display improved activity, with lower IC_50_ values compared to those of CLR01. Suppression of infection was dose-dependent for all three endemic human coronaviruses hCoV-NL63, -229E, and -OC43 ^48–50^ (Figures 6a,b and S17). For ZIKV, which buds from the ER,^31,32^ and HSV-1 and HSV-2, which bud from the Golgi apparatus^33^ advanced tweezers, particularly those carrying C6/C7 alkyl or aromatic groups, also showed enhanced activity (Figures 6a, S17, and 18).

Principal component analysis (PCA) of all IC_50_ and EC_50_ values confirmed that the budding site and envelope composition influence susceptibility. Coronaviruses, except hCoV-OC43, clustered together, while PM-budding viruses grouped separately near virus-like liposomes (Figure 6b). This indicates that tweezer activity differs between vesicles and GUVs, and is affected by both lipid composition and vesicle size. To exclude cell line effects, hCoV-OC43 was also propagated in HCT-8 cells, yielding IC_50_ values comparable to those obtained with TMPRSS2-expressing Vero E6 cells (Figure S19). A second PCA correlating tweezer activity with individual lipid liposomes showed that phospholipid vesicles (PA, PE, PS, PI, PG), but not PC, clustered with coronaviruses, suggesting that headgroup chemistry is a major determinant of susceptibility (Figure 6c). In contrast, PM-, ER-, and Golgi-budding viruses localized near PC, PC/SM, and lyso-lipid liposomes, while PC/Chol liposomes clustered distantly, consistent with their lower susceptibility. Together, these findings support the concept that the site of viral budding and, thus, the lipid composition of the envelope are critical factors shaping the antiviral efficacy of molecular tweezers.

## Discussion

Broad-spectrum antivirals are highly desirable for preparedness against emerging and challenging viral pathogens. In contrast to protein-targeting drugs, which are prone to viral escape, agents that attack the viral membrane act on a highly conserved and indispensable structure. MTs represent such compounds. The prototype CLR01 was originally developed as an antiamyloid compound and has been extensively studied for its ability to remodel pathological protein aggregates.^9,51^ Later, CLR01 was found to display antiviral activity by disrupting the lipid envelopes of a broad range of viruses, including HIV-1, herpesviruses, influenza virus, Ebola virus, Zika virus, and SARS-CoV-2.^9,15–17^ Mechanistic studies showed that CLR01 and CLR05 bind to choline headgroups of PC and SM, forming inclusion complexes that induce lipid orientation changes and destabilize viral membranes.^15,18^ These “parental” tweezers provided the foundation for the development of advanced derivatives.

Building on these findings, we recently introduced ester-functionalized MTs carrying aliphatic or aromatic substituents, which serve as lipid anchors.^15,18^ These advanced tweezers displayed markedly increased potency in cell culture, airway mucus, and animal models.^18^ Combined with previous work,^18^ the present study now provides the mechanistic explanation for this enhanced activity. We demonstrate that advanced MTs retain the ability to bind choline headgroups but additionally act through hydrophobic insertion of their ester arms into membranes, allowing them to target noncholine lipids. This dual mechanism expands the lipid range susceptible to tweezers and explains the broad-spectrum antiviral efficacy. MD simulations further suggest a dynamic process in which tweezers can work through mechanisms involving repeated insertion into the membrane (by the same or a different tweezer molecule) as well as diffuse laterally along the membrane, a “lawn mower-like” mode of action.

The lipid specificity of advanced tweezers was investigated to understand how the membrane composition influences the antiviral activity. Liposome leakage experiments revealed that the presence of sphingomyelin (SM) and unsaturated phospholipids strongly enhanced tweezer-induced membrane disruption, while cholesterol- or saturated-phosphatidylcholine (PC)-rich membranes were more resistant. These findings indicate that membrane order and fluidity critically determine the susceptibility. Fluid membranes with high lateral mobility and packing defects allow deeper insertion of the tweezer’s aromatic arms, whereas tightly packed and rigid bilayers formed by cholesterol and saturated PC hinder this process.^39,41^

Membrane curvature also influenced the tweezer activity. Smaller, highly curved vesicles were more efficiently disrupted than larger ones, consistent with the enhanced insertion into strained bilayers. When normalized for lipid content, advanced tweezers were approximately 260-fold more active on 100 nm virus-like liposomes than on giant unilamellar vesicles (GUVs), suggesting that curvature facilitates local bilayer perturbation. This curvature dependence provides a mechanistic explanation for the high susceptibility of small enveloped viruses such as ZIKV. Importantly, the lower curvature and higher cholesterol content of cellular plasma membranes may explain the low cytotoxicity of advanced MTs, as these features hinder tweezers insertion and promote membrane stability. In addition, cells can rapidly reseal transient membrane defects via active repair mechanisms,^52,53^ whereas viruses lack such machinery, rendering tweezer-mediated damage irreversible.

To further dissect the origin of these effects, we examined whether the viral budding site, and thus the envelope lipid composition, affects the MT activity. Viruses that bud from lipid-raft-enriched plasma membranes (HIV-1, RSV, MeV)^25–27^ were highly sensitive to MTs, consistent with their enrichment in SM and cholesterol.^45–47^ In contrast, SARS-CoV-2, which buds from the ERGIC,^28–30^ showed distinct susceptibility patterns, reflecting the different lipid environment of its envelope. Testing of other ERGIC-budding coronaviruses (hCoV-NL63, -229E, and -OC43)^28–30^ confirmed this relationship: advanced tweezers exhibited stronger inhibition with lower IC_50_ values compared to CLR01, and the suppression of infection was dose-dependent. Similarly, ZIKV (ER-budding)^31,32^ and HSV-1/-2 (Golgi-budding)^33^ were inhibited, particularly by C6/C7 alkyl and aromatic tweezers.

Principal component analysis (PCA) integrating IC_50_ and EC_50_ values from viral and liposome assays further underscored these relationships. Coronaviruses, except hCoV-OC43, clustered together, whereas PM-budding viruses grouped separately near virus-like liposomes. This indicates that the tweezer activity is shaped by both the lipid composition and particle curvature. To exclude potential cell-line-specific effects, hCoV-OC43 propagated in human HCT-8 cells showed similar IC_50_ values to virus grown in TMPRSS2-expressing Vero E6 cells. A second PCA comparing individual lipid liposomes revealed that phospholipid vesicles (PA, PE, PS, PI, PG), but not PC, clustered closely with coronaviruses, indicating that headgroup chemistry contributes to susceptibility. PM-, ER-, and Golgi-budding viruses were located near PC, PC/SM, and lyso-lipid liposomes, while PC/Chol liposomes formed a distant cluster consistent with reduced tweezer sensitivity.

While the potent membrane activity of MTs offers clear advantages, certain limitations must be considered for clinical translation. Previous studies demonstrated that the antiviral activity of CLR01 and related MTs is reduced in the presence of serum proteins, likely due to sequestration of the tweezers by serum components.^16,17^ This property may limit systemic application as free compound concentrations sufficient to disrupt viral envelopes would be difficult to achieve in circulation without off-target binding. Nevertheless, the strong antiviral efficacy, low cytotoxicity, and high selectivity for viral membranes identified here highlight MTs as highly promising candidates for topical or localized administration. Their dual mechanism, combining choline headgroup binding with hydrophobic insertion, renders MTs broadly active against respiratory viruses such as SARS-CoV-2, common cold corona viruses, influenza, and RSV, which replicate on airway surfaces, where serum inactivation is minimal. Similarly, the ability to target SM- and unsaturated-lipid-rich membranes supports their potential use in topical treatment of HSV-1 and HSV-2 lesions, which are accessible for direct application. Formulation as nasal sprays, inhalable aerosols, or antiviral creams could therefore enable preventive or early therapeutic intervention while minimizing systemic exposure.

Beyond these applications, the broad antiviral spectrum, low risk of resistance, and unique host-targeting mechanism of MTs highlight their potential role in pandemic preparedness and protection against future emerging pathogens. By targeting the conserved lipid envelopes of diverse viruses, advanced MTs could serve as rapidly deployable mechanism-based countermeasures in early outbreak scenarios. Together, these properties make molecular tweezers a promising new class of membrane-targeting antivirals with considerable potential for both clinical and public health impact.

## Supplementary Material



## Data Availability

All primary data will be made accessible to qualified researchers upon reasonable request.

## Notes

Material and methods, supplementary figures, and references (PDF)
