# Supplementary material for: Advanced Molecular Tweezers Effectively Target Membranes Lacking Choline Headgroups for Broad-Spectrum Antiviral Efficacy
Source: J Am Chem Soc. 2026 Jan 19;148(3):3626–37. doi: 10.1021/jacs.5c19450 (PMC12856888; doi:10.1021/jacs.5c19450)
Supplement: Supplementary file 1 [file ja5c19450_si_001.pdf]

Supporting Information

Advanced Molecular Tweezers Effectively Target Membranes Lacking Choline Headgroups for Broad-Spectrum Antiviral Efficacy

Tatjana Weil*[1], Jan Lawrenz*[1], Estelle Taghuo Kaptouom[2], Joel Mieres-Perez[3], Victoria Hunszinger[1], Konstantin M. J. Sparrer[1,4], Yasser Almeida-Hernandez[3], Thomas Schrader[2], Elsa Sanchez-Garcia[3#] and Jan Münch[1#]

[1] Institute of Molecular Virology, Ulm University Medical Center, 89081 Ulm, Germany
[2] Faculty of Chemistry, University of Duisburg-Essen, 45117 Essen, Germany
[3] Chair of Computational Bioengineering, Department of Biochemical and Chemical Engineering, TU Dortmund University, 44227 Dortmund, Germany
[4] German Center for Neurodegenerative Diseases (DZNE), 89081 Ulm, Germany

*Contributed equally
# Corresponding author: jan.muench@uni-ulm.de; elsa.sanchez@tu-dortmund.de

Supplementary Material and Methods: pages 1-6

Supplementary Figures pages 7-25

Supplementary References pages 26-27

Supplementary Material and Methods

Synthesis and characterization of dialkyl diphosphate tweezers. Molecular Tweezers were synthesized and purified according to the method described previously[1].

Cell culture. Caco2 cells (kindly provided by Prof. Barth, Ulm University) were grown in Dulbecco's modified Eagle's medium (DMEM) supplemented with 10% heat-inactivated fetal calf serum (FCS), 100 units/ml penicillin, 100 µg/ml streptomycin, 2 mM L-glutamine, 1 mM sodium pyruvate, and 1x non-essential amino acids (NEAA). Vero E6 (African green monkey derived epithelial kidney; ATCC® CRL-1586TM) cells were grown in the same medium as Caco2 cells, but with 2.5% FCS. Huh-7 (hepatocyte-derived carcinoma; kindly provided by Anna-Laura Kretz, Department for General and Visceral Surgery, Ulm University) were grown in DMEM supplemented with 10% heat-inactivated FCS, 100 units/ml penicillin, 100 µg/ml streptomycin and 2 mM L-glutamine. ELVIS cells (enzyme-linked virus-inducible system cells; hamster kidney cells and purchased from ATCC®) were cultured under the same conditions as Huh-7 cells. TMPRSS2-expressing Vero E6 cells (kindly provided by the National Institute for Biological Standards and Control (NIBSC), #100978) were cultivated in DMEM supplemented with 10 % heat-inactivated FCS, 2 mM L-glutamine, 100 U/ml penicillin, 100 mg/ml streptomycin and 1 mg/ml

geneticin. LLC-MK-2 (rhesus monkey kidney; kindly provided by Lia van der Hoek, Amsterdam, The Netherlands) were grown Eagle's minimal essential medium (MEM) supplemented with 100 units/ml penicillin, 100 µg/ml streptomycin, 2 mM L-glutamine, 1x NEAA and 8 % heat-inactivated FCS. HCT-8 cells (human ileocecal adenocarcinoma; obtained from ATCC®) were cultivated in Rosewell Park Memorial Institute Medium (RPMI-1640) supplemented with 10% heat-inactivated FCS, 100 units/ml penicillin and 100 µg/ml streptomycin. Cells were grown at 37°C in a 5% CO2 humidified incubator. All cell lines were tested on a regular base for mycoplasma contamination and remained negative.

hCoV NL63, 229E and OC43 stock. HCoV-229E (ATCC® VR-740™) was propagated on Huh-7 cells, - NL63 (Netherland 63) on LLC-MK2 cells (kindly provided by Lia van der Hoek, Amsterdam, The Netherlands), and -OC43 (Organ Culture 43; ATCC® CR-1558 ™) on TMPRSS2-expressing Vero E6 cells or HCT-8 cells. To this end, 80% confluent cells in a T-75 flask were inoculated with a multiplicity of infection (MOI) of 0.1 in 10 ml medium supplemented with 2% FCS and kept at 33 °C. One day post inoculation cells were washed with PBS before 10 ml of fresh medium supplemented with 2% FCS was added. The following days, cells were monitored daily under the light microscope until a strong cytopathic effect (CPE) was visible (3 days for hCoV-229E, 5 days for hCoV-NL63 and 4 days for hCoV-OC43). Virus stocks were then harvested by centrifugation of the supernatant for 5 min at 1300 rpm to remove cellular debris and storing aliquots at - 80 °C.

ZIKV stock: The Asian ZIKV strain FB-GWUH-2016 were isolated from a fetal brain with severe abnormalities[2] and kindly provided by Olli Vapalahti, Department of Virology, University of Helsinki. For propagation, a T-75 flask with 70 % confluent Vero E6 cells was inoculated with ZIKV after renewal of the medium with 10 ml medium supplemented with 25 mM HEPES. 2 h post-inoculation, further 15 ml HEPES supplemented medium was added. Cells were monitored daily until strong CPE was visible. On day 3, cell supernatant with detached cells was harvested, cleared by centrifugation at 325 x g for 3 min and supernatant stored at - 80 °C.

HSV-1 and HSV-2 stock: Recombinant eGFP-encoding Herpes-Simplex-Virus 2 (strain 333; kindly provided by Patricia Spear; Northwestern University, USA) and HSV-1-GFP (strain F; kindly provided by Benedikt Kaufer; Free University of Berlin) were propagated on Vero E6 cells. In brief, 70 % confluent Vero E6 cells in T-75 flask were inoculated with HSV-1-GFP or HSV-2-GFP at a MOI of 0.1, after medium was refreshed. Cells were incubated until strong CPE was visible at day 2. Virus was harvested by centrifugation at 1500 rpm for 5 min to remove cellular debris and storing aliquots at - 80 °C.

hCoV 229E, -NL63 and -OC43 inhibition assay. 25,000 Caco2 cells (for h-CoV-NL63) or 25,000 Huh-7 cells (in case of hCoV-229E and -OC43) were seeded in 100 µl medium in a 96 well plate. The next day 44 µl of medium were added on cells. Afterwards, 70 µl serial titration of tweezer was mixed with 70 µl hCoV-229E, -NL63 or -OC43 for 30 min at 33°C. 36 µl of the tweezer-virus mix was then added on cells, resulting in an MOI of 0.01 (hCoV-NL63), 0.002 (hCoV-229E) or 0.006 (hCoV-OC43) and incubated at 33 °C. Infection rates were assessed at 2 days (hCoV-229E), 3 days (hCoV-OC43) or 6 days (hCoV-NL63) post infection by in-cell ELISA detecting nucleocapsid[3]. Briefly, cells were fixed by addition of 180 µl 8 % paraformaldehyde (PFA) for 30 min at room temperature (RT) and permeabilized by incubation in 100 µl 0.1% TritonX-100 for 5 min. After washing once with PBS, cells were stained with 50 µl of 1:5,000 diluted anti-nucleocapsid protein antibody (Sino Biologicals; anti-229E (40640-T62); anti-NL63 (40641-T62); anti-OC43 (40643-T62)) in antibody buffer (10% FCS and 0.3% Tween-20 in PBS) for 1 h at 37°C. Subsequently, cells were washed twice in washing buffer (0.3% Tween-20 in PBS) and incubated for additional hour at 37 °C in the secondary HRP-conjugated antibody (Thermo Fisher #31460) (1:15,000 in case of hCoV-229E and 1:10,000 for hCoV-NL63 and -OC43 in antibody buffer). Following three times of washing with washing buffer, 50 µl of the TMB peroxidase substrate (Medac

#52-00-04) was added for 5 min and the reaction was stopped using 50 µl 0.5 M H_2SO_4. Optical density (OD) was recorded at 450 nm with 620 nm background correction - using an ELISA microplate reader. Signal derived from uninfected cells was subtracted and untreated controls were set to 100% infection.

ZIKV inhibition assay. To determine inhibition rate of ZIKV by tweezers, 6,000 Vero E6 cells were seeded in 100 µl respective medium. After overnight incubation, 60 µl medium was added on cells and an 80 µl tweezer prepared dilution series was mixed with 80 µl ZIKV strain FB-GWUH-2016, followed by incubation for 30 minutes at 37°C. Afterwards, cells were inoculated with 40 µl tweezer-virus mix (MOI 0.15). Infection rates were assessed after 48 h by detection of flavivirus E protein as described[4]. In brief, supernatant was removed, cells were washed once with PBS and fixed with 100 µl 4 % PFA for 20 min at RT. After aspiration of PFA, cells were permeabilized with ice cold methanol for 5 min and further washed with PBS. Subsequently, cells were stained for 1 h at 37 °C with 50 µl of 1: 10,000 diluted mouse anti-flavivirus protein E antibody 4G2 (Absolute Antibody #Ab00230-2.0) in antibody buffer (10% FCS and 0.3% Tween-20 in PBS) and washed three times with washing buffer (0.3% Tween-20 in PBS). 50 µl of 1: 20,000 diluted secondary anti-mouse antibody conjugated with HRP (Thermo Fisher #A16066) was added on cells and incubated for further 1 h at 37 °C followed by four times of washing with washing buffer. TMB peroxidase substrate (Medac #52-00-04) was added for 5 min and the reaction was stopped using 0.5 M H_2SO_4. OD was recorded at 450 nm with 620 nm background correction - using an ELISA microplate reader. Signal derived from uninfected cells was subtracted and untreated controls were set to 100% infection.

HSV-1 and HSV-2 inhibition assay. For measurement of tweezer inhibition rates against HSV-1 and HSV-2, 10,000 ELVIS cells (encode a lacZ gene which is expressed upon infection via the trans-activator ICP10[5]) were seeded in 100 µl medium. At the next day, medium was replaced by 144 µl X-VIVO 15 (Lonza; #BE02-060F) supplemented with 100 units/ml penicillin, 100 µg/ml streptomycin, 2 mM L-glutamine. A serial dilution of tweezers was incubated either with 70 µl of HSV-1 or HSV-2 for 15 min at 37 °C, before 36 µl tweezer-virus mix was used for infection of cells, resulting in a MOI of 0.05. Two days post infection, infection rates were determined by detecting β-galactosidase activity in cellular lysates using the Tropix Gal-Screen kit (Applied Biosystems) and the Orion microplate luminometer (Berthold) for measurements. Values represent β-galactosidase activities (relative light units per second; RLU/s), with subtracted background of uninfected cells and normalization to infected cells in absence of tweezer´s.

Biomolecular modelling. The simulation systems comprise the six possible combinations of the tweezers CP002 and CP024 with the three membranes POPG, POPA and POPS. All the systems were prepared using the CHARMM-GUI Membrane Builder[6,7]. The systems were composed by 128 units of POPG, POPA, or POPS phospholipids and five tweezer units (CP024, CP002) initially placed manually on top of the upper leaflet of the membrane at a distance of 4 Å from the closest lipid's phosphorous atom in the normal of the membrane. The CHARMM general force field (CGenFF) parameters[6] were used for the tweezers. Parameters for molecular tweezers obtained employing CGenFF, have been tested and used by us in previous studies[8,9]. The lipid bilayers have a dimension of 10 × 10 nm. The systems were solvated using the TIP3P water model[10], and 0.15 mM NaCl were added. After minimization and equilibration, the systems were simulated for 1 µs each time (3 replicas of 1 µs each per system) using GROMACS[11] and the CHARMM36m force field[12]. Long-range electrostatic interactions were treated with the Particle Mesh Ewald (PME) method[13]. Short-range Lennard-Jones and electrostatic interactions were calculated with a switching function between 10 Å and a cut-off value of 12 Å. The temperature was maintained at 300 K using Langevin dynamics[14] and the Nose–Hoover piston method was employed for keeping the pressure constant at 1 atm[15]. To favour the interaction of the tweezers only occurs with the lipids in the upper leaflet, flat-bottom positional restraints were applied

to the heavy atoms of the tweezers, from a distance of 5 nm and a force of 1000 kJ/mol.nm^2. To evaluate the orientation of the tweezers with respect to the membrane, we calculated the angle µ (Supplementary Scheme S1) between the vector formed by the core carbon atoms of the tweezer (C1 and C2 for CP002 and C1 and C for CP024) and the Z-axis of the simulation box, using the tool *gmx gangle* of the GROMACS[11] suite.

The binding of the tweezers to the membranes was characterized as follows (Figs. S6-S8): For each frame of the trajectory, the maximum Z-coordinate of the box (Z_{max}) and the Z-coordinate of the centre of mass (Z_{COM}) of each tweezer was measured in order to characterize the tweezer´s position in the Z-axis. A binding event is considered to take place when the tweezer penetrates the upper boundary of the membrane defined by the phosphorous atoms of the lipids surrounding the tweezer in the upper leaflet. In the case of the tweezer 14E with the POPS membrane, we saw that in one replica, sometimes the tweezer binds the lower leaflet, despite the position restraints applied. In order to normalize this binding to the rest of the systems, the following logic was applied. If the value of the $Z_{COM} > 4$ nm, the value remains the same. If $Z_{COM} < 2$ nm, then $Z_{COM} = Z_{max} + Z_{COM}$, if $2 \leq Z_{COM} \leq 4$ nm, then $Z_{COM} = Z_{max} - Z_{COM}$.

NMR titrations. All NMR-titrations were carried out in methanol-d_4 at a temperature of 25°C. A stock solution of the lipid guest (SM, DOPC) was prepared by dissolving it in methanol-d4 at a concentration of 0.33 mM and 600 µL of this solution were filled in an NMR tube for the first 1H-NMR spectrum measurement. The tweezers host solution was prepared at a concentration of 10 mM using the lipid guest solution to keep the guest concentration constant throughout the titration. During the titration, the host solution was added in increasing amounts (10-100 µL), resulting in effective host concentrations of 0.16 mM, 0.32 mM, 0.48 mM, 0.63 mM, 0.91 mM, 1.18 mM, 1.43 mM, 1.89 mM, 2.31 mM, 2.86 mM, 3.33 mM and 4.00 mM. The concentration of the host was gradually increased until a final host/guest ratio of about 10:1. NMR titrations were carried out on the Bruker Advance DRX 500 (500 MHz) at 25°C. The NMR spectra were acquired immediately after each addition of the host solution. The spectra were processed using the MestReNova software. The chemical shift changes of the N(Me)3+ protons were monitored during the titration to determine the binding constants. The binding isotherms were generated by plotting the chemical shift changes as a function of the host concentration. Non-linear regression was performed to calculate the 1:1 affinity and Δδmax values.

Surface Tension Measurements. 1,2-Dioleoyl-sn-glycero-3-phosphocholine (DOPC) and Sphingomyelin (SM) were purchased from *Avanti Polar Lipids* with purity degree higher than 99%, and dissolved in chloroform at a concentration of 1 mg/mL. Molecular tweezers were synthesized and purified according to the method described previously[1]. Tweezers solutions were prepared as 1mg/mL solutions in PBS buffer. Surface pressure vs. area curves were obtained using a Langmuir-Blodgett Trough Small-2 with an area of 84 cm^2. The instrument employs the Wilhelmy method with platinum wire probe attached to the microbalance sensor head. To perform the experiments, the Langmuir trough was filled with PBS buffer as subphase. The subphase volume was maintained at 70 mL. Subsequently, 20 µL of DOPC or sphingomyelin (SM) or a mixture of both dissolved in Chloroform was spread in several tiny drops on the buffer surface using a Hamilton micro syringe. The lipids were allowed to stabilize for a sufficient period to ensure complete evaporation of the chloroform. A 20 µL solution of the tweezer (1 mg/mL) was then slowly injected into the backside of the barrier, so as not to disturb the lipid monolayer. After waiting for approximately 5 min, the molecular film was compressed, and a p/A isotherm was recorded at a compression rate of 10 cm^2/min. To maintain the integrity of the experiments, the equipment was enclosed in an acrylic box to prevent contamination from the environment.

Liposome production. Liposomes were prepared by thin-film hydration and extrusion. Virus-like liposomes consisting of 18:1(cis) PC / DOPC, sphingomyelin (Egg SM) and cholesterol (ovine wool; all lipid purchased at Avanti Polar Lipids) were mixed at 45/25/30 mol% ratio in a glass flask with a lipid concentration of 5 mM[16]. For preparation of liposomes consisting of single or double lipid source, the following lipids were used and mixed with the same concentration in a glass flask: PC (phosphatidylcholine; Egg PC), PE (phosphatidylethanolamine; Egg PE), PS (phosphatidylserine; Brain PS), PI (phosphatidylinositol; Soy PI), PA (phosphatidic acid; Egg PA), PG (phosphatidylglycerol; Egg PG), sphingomyelin (Egg SM), ceramide (Egg Cer), Chol ester (cholesteryl oleate; 18:1), cholesterol (ovine wool), lyso-PC (1-oleoyl-2-hydroxy-sn-glycero-3-phosphocholine), lyso-PS (1-oleoyl-2-hydroxy-sn-glycero-3-phospho-L-serine), lyso-PE (1-oleoyl-2-hydroxy-sn-glycero-3-phosphoethanolamine, 18:0 PC / DSPC (1,2-distearoyl-sn-glycero-3-phosphocholine)¸ 18:1(cis) PC / DOPC (1,2-dioleoyl-sn-glycero-3-phospho-choline), 18:2 (cis) PC / DLPC (1,2-dilinoleoyl-sn-glycero-3-phosphocholine) and 18:3 (cis) PC (1,2-dilinolenoyl-sn-glycero-3-phosphocholine), all lipid purchased at Avanti Polar Lipids). Liposomes consisting out of two lipid sources were mixed at a ratio of 50/50 mol% or in case of lyso-lipids at a ratio of 95/5 mol% ratio. Chloroform as solvent was evaporated by applying nitrogen stream and the resulting lipid film was hydrated in an iso-osmolar 50 mM 5(6)-carboxyfluorescein PBS/water solution, adjusted to pH of 7.4 with NaOH. The glass vials were shacked at 60 °C with agitation of 160 rpm for 1h. Liposomes were prepared by at least 20x extrusion through polycarbonate membrane (Nuclepore Track-Etced Membrane, Whatman) ranging from 0.05 to 0.8 µm pore size in a Mini Extruder (Avanti Polar Lipids), placed on a heating platform at 60 °C. Non-encapsulated dye was removed with size-exclusion filtration using PD midiTrap Sephadex G-25 columns (Cytiva), performed twice. Liposomes were characterized by nanoparticle tracking analysis (NTA) using Zeta View TWIN (Particle Metrix, Inning, Germany) or dynamic light scattering (DLS) using Zeta Sizer Nano (Malvern Panalytical). In case of NTA measurements, samples were diluted in PBS and videos of the scattering particles were recorded with the following settings: 25 °C, 11 positions, 1 cycle, sensitivity 85 – 90, shutter 100, 15 fps, 2 s videos/positions, 3 – 5 measurements. Between the samples, the chamber was flushed with PBS. For DLS, samples were diluted in PBS and measured in a cuvette with automated settings for attenuator and position. 3 independent acquisitions were performed per sample.

GUV production and characterisation. Virus-like giant unilamellar vesicles (GUV´s) were produced based on previous publication[17]. Briefly, lipids were mixed in a glass flask at a final concentration of 5 mM and applied on Whatman Grade 1 paper (Cytiva #1001-070) sandwiched between CellCrown 24 (Scaffdex) inserts in a 24 well plate. Chloroform as solvent was evaporated by applying nitrogen stream and lipid film was re-hydrated in 1.5 ml isoosmolar 50 mM 5(6)-carboxyfluorescein PBS/water solution, adjusted to pH of 7.4 with NaOH. The plate was shacken at 60 °C with agitation of 160 rpm for 1h. For further detachment of the GUV´s, membrane was puffed with an empty pipet tip and free-dye was removed by size-exclusion filtration using PD midiTrap Sephadex G-25 columns (Cytiva), performed twice. Size and concentration were measured using Luna II Cell Counter (Logos Biosystems) at three positions.

Dye leakage assay. Tweezer activity against liposomes or GUV´s was tested in dye leakage assay. To this end, 90 µl liposomes or GUV´s were added into 96-well plate. Baseline was generated by measuring fluorescence at excitation 485 nm and emission at 528 nm for 5 min in a Synergy H1 plate reader (BioTek), before 10 µl tweezer solution in escalating concentrations was added and fluorescence recorded for 30 min with measurement every minute. Maximum dye leakage was achieved by addition of Triton X-100 at a final concentration of 1 % and measured for 5 min. Background signals were subtracted from measured values, and normalized to maximum fluorescence. Area under the curve was calculated from each concentration and potted accordingly.

Principal component analysis (PCA). PCA was conducted using R version 4.1.2[18]. In brief, IC_{50} and EC_{50} values of tweezers against viruses or liposomes were used for PCA. To account for the impact of missing values and enable comparison of the different tweezers, viruses and synthetic vesicles, missMDA package version 1.18[19] was used to impute the missing values and remove their impact on the PCA. PCA was done using the factoMineR package version 2.4[20] and results were extracted using the facto extra package version 1.0.7[21]. The first two dimensions were plotted with the tidyverse package collection[22] using the extracted dimensions.

Non-linear regression and statistics. Unless stated otherwise, analysis was performed using GraphPad Prism version 10.0.2. IC_{50} or EC_{50} values were calculated with a non-linear regression model (inhibitor vs. normalized response, variable slope). Correlation analysis was assessed by Spearman Correlation, two-tailed p value. Further statistical analysis was performed with nonparametric Mann-Whitney test. Chemical structures were realized with ChemDraw version 20.0.

Supplementary Figures

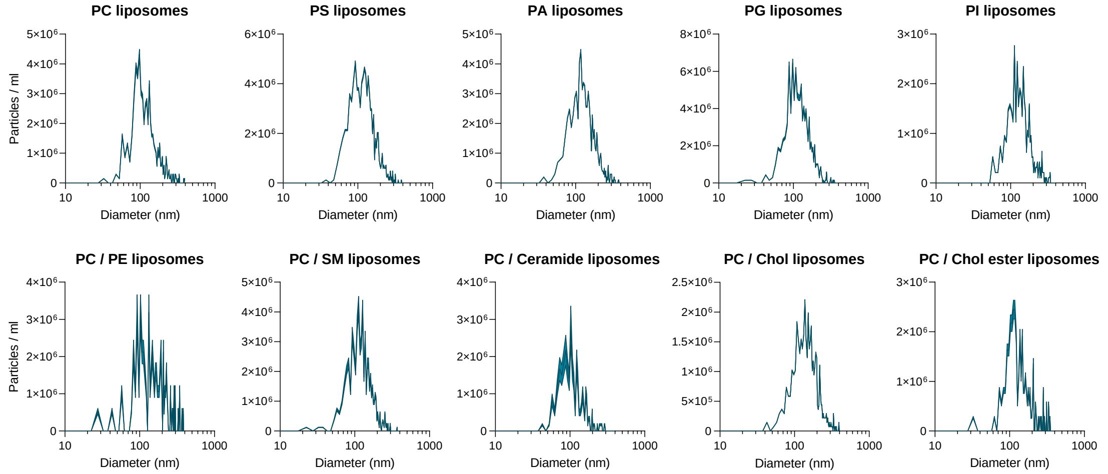

Fig. S1. Characterisation of single / double sourced lipid liposomes. a, NTA of each liposome type consisting of one or two lipid sources. Measurement was conducted in 3 acquisitions and shown as mean ± SD in shaded line. Modified from Weil, 2023[23] (CC BY 4.0; https://creativecommons.org/licenses/by/4.0/).

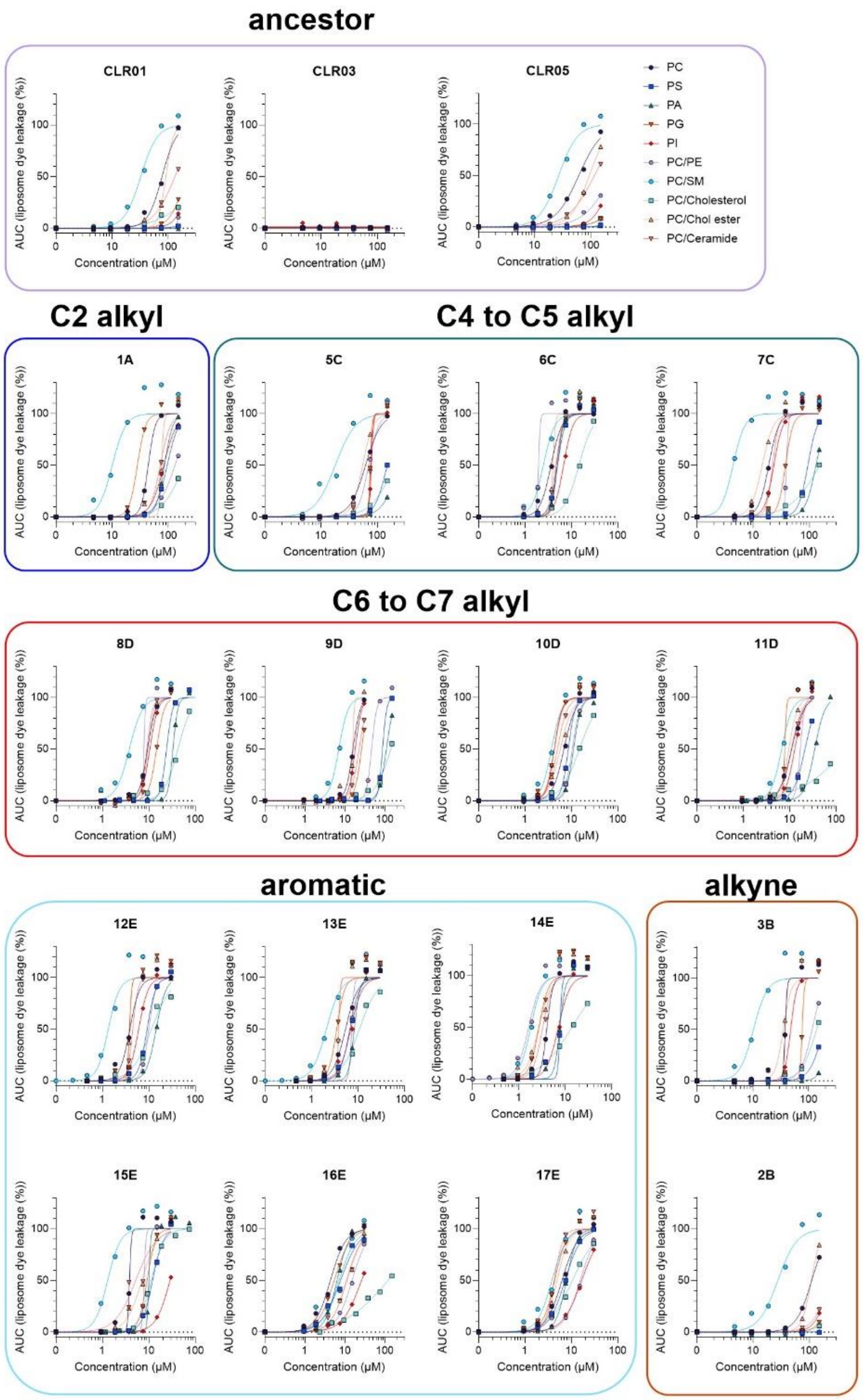

Fig. S2. Advanced MTs demonstrate a broad membranolytic capacity across various lipid compositions. Liposomes (2.5 x 10^{10} particles/ml) were incubated with increasing concentrations of tweezers for 30 min and fluorescence was recorded every minute at 485 nm excitation and 528 nm emission. Baseline was measured for 5 min in absence of tweezers and maximum fluorescence was recorded after the addition of 1 % Triton X-100. Values were corrected for baseline and normalized to maximum fluorescence. Graphs show area under the curve in means of one experiment performed in triplicates. Modified from Weil, 2023[23] (CC BY 4.0; https://creativecommons.org/licenses/by/4.0/).

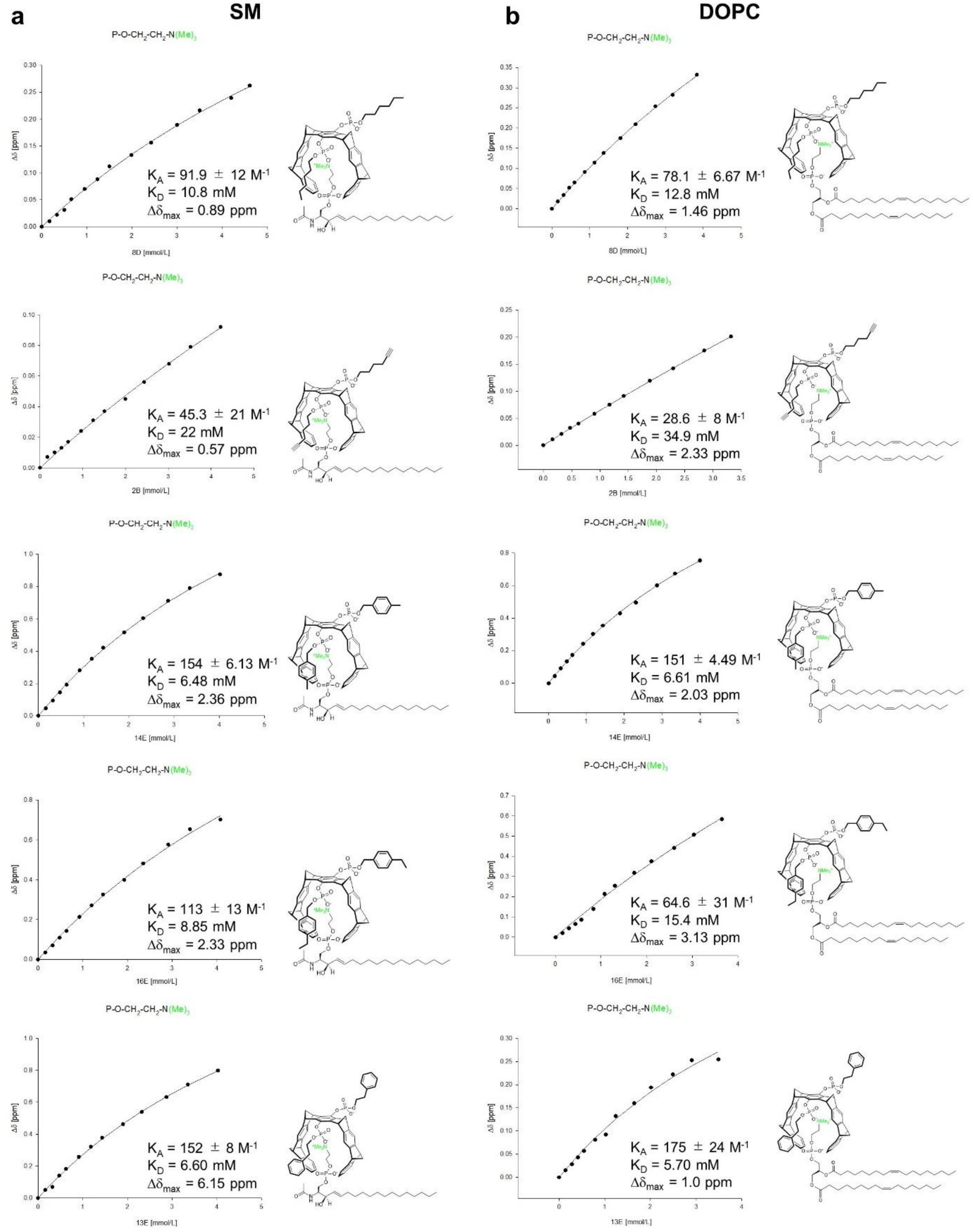

Fig. S3. ^{1}H-NMR titrations in methanol-d_4 between advanced molecular tweezers 8D, 2B, 14E, 16E and 13E and SM or DOPC. Binding curves of 8D, 2B, 14E, 16E, 13E for the $N(Me_3)^+$ group with the resulting affinity (K_A/K_D) and Δδmax value obtained from nonlinear regression and corresponding Lewis structure showing inserted lipid head group of Sphingomyelin (**a**) or DOPC (**b**) inside the cavity of the tweezers.

a **b**

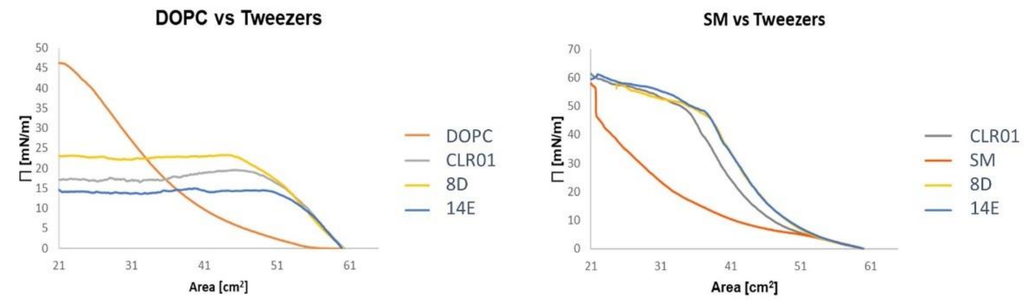

Fig. S4. Characterization of the interaction between molecular tweezers and lipid monolayers spread on PBS-Puffer. Surface tension/area curves were obtained for monolayers from DOPC (**a**) and SM (**b**) prior and after subinjection of CLR01 or 8D or 14E. Tweezer subinjection leads to a considerable area increase and steeper slopes of compression curves, indicating tweezer incorporation into the monolayer and increased surface pressure. At much lower pressure thresholds, the curves lean back and indicate disruption of the monolayer. Advanced tweezers 8D and 14E produce stronger effects than their ancestor tweezer CLR01.

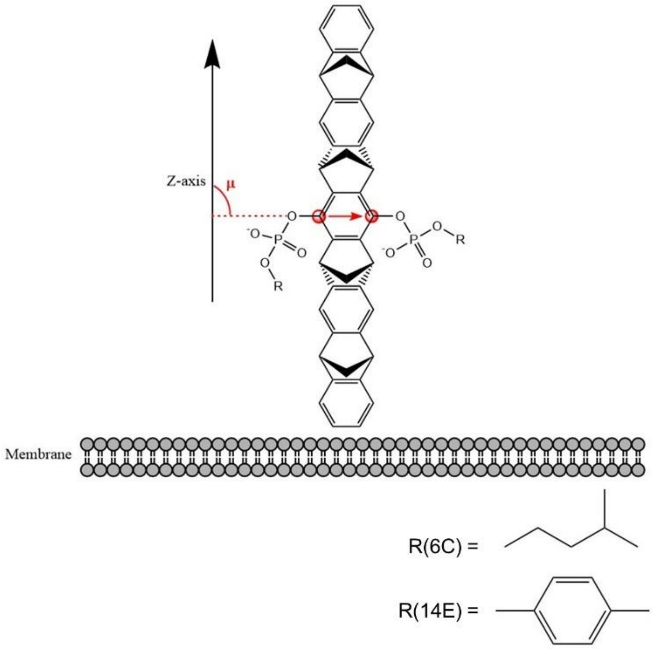

Fig. S5. Tweezer orientation with respect to the membrane. To characterize the orientation of the tweezer, the angle μ was measured during the simulation. The μ angle comprises the vector formed by the carbon atoms attached to phosphate groups (red arrow) and the Z-axis of the simulation box, normal to the plane of the membrane. R groups correspond to specific chemical moieties in each tweezer.

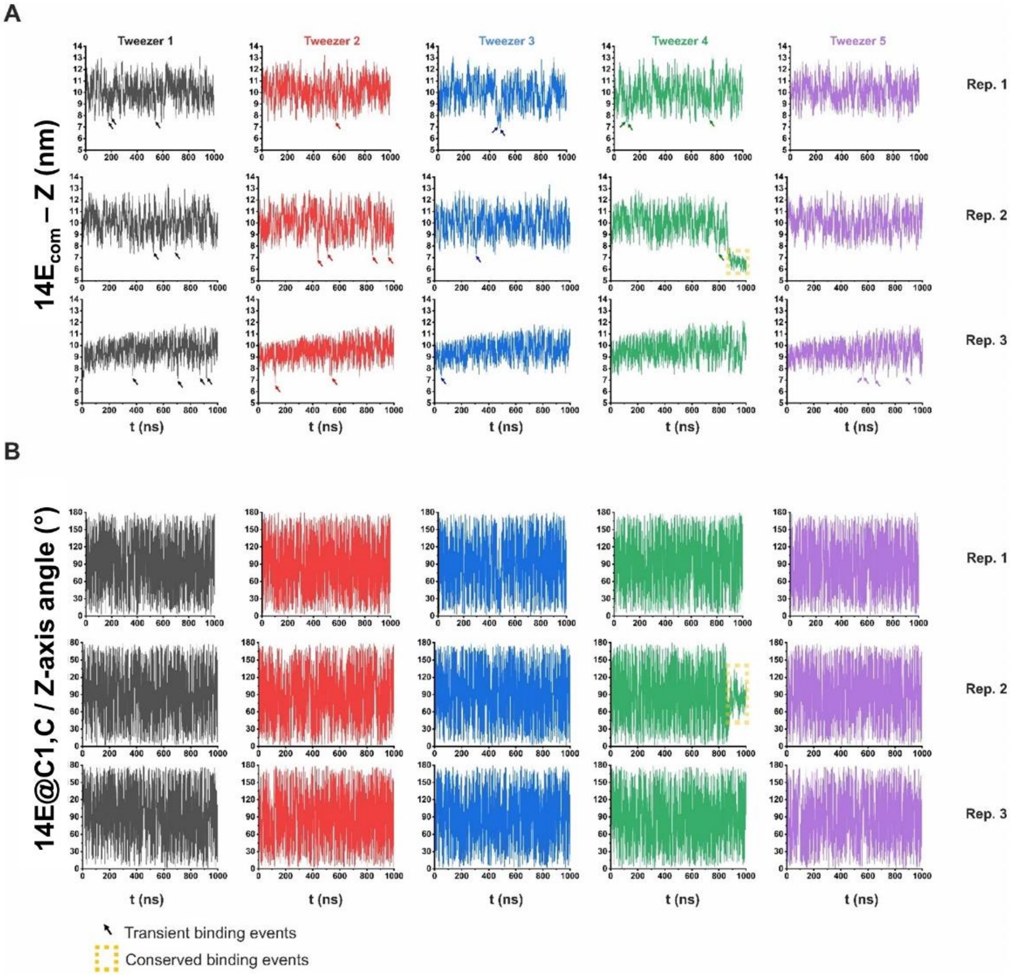

Fig. S6. A) Binding events of 14E with the upper leaflet of the POPG membrane during the MD simulations. The arrows mark transient binding events while the yellow dotted box indicates the conserved binding event. Each row shows the results per replica (Rep. 1, Rep. 2, or Rep. 3) for each tweezer molecule (columns). The Y-axes correspond to the Z-coordinate of the center of mass of the tweezer.

Due to the dynamics of the membrane bilayer and the different orientation of the tweezer with respect to the membrane, the relative positions (with respect to the tweezer) of the head groups of the lipids change during the simulation and are therefore not shown. Instead, we visualized the trajectories to follow the binding events. A binding event is considered to take place when the tweezer penetrates the upper boundary of the membrane, defined by the phosphorous atoms of the lipids surrounding the tweezer in the upper leaflet. The same analysis was applied in Figs. S7 and S11.

B) Tweezer orientation with respect to the membrane. Time evolution of the angle μ (Fig. S5) during the simulation. The yellow dotted box indicates the conserved binding event.

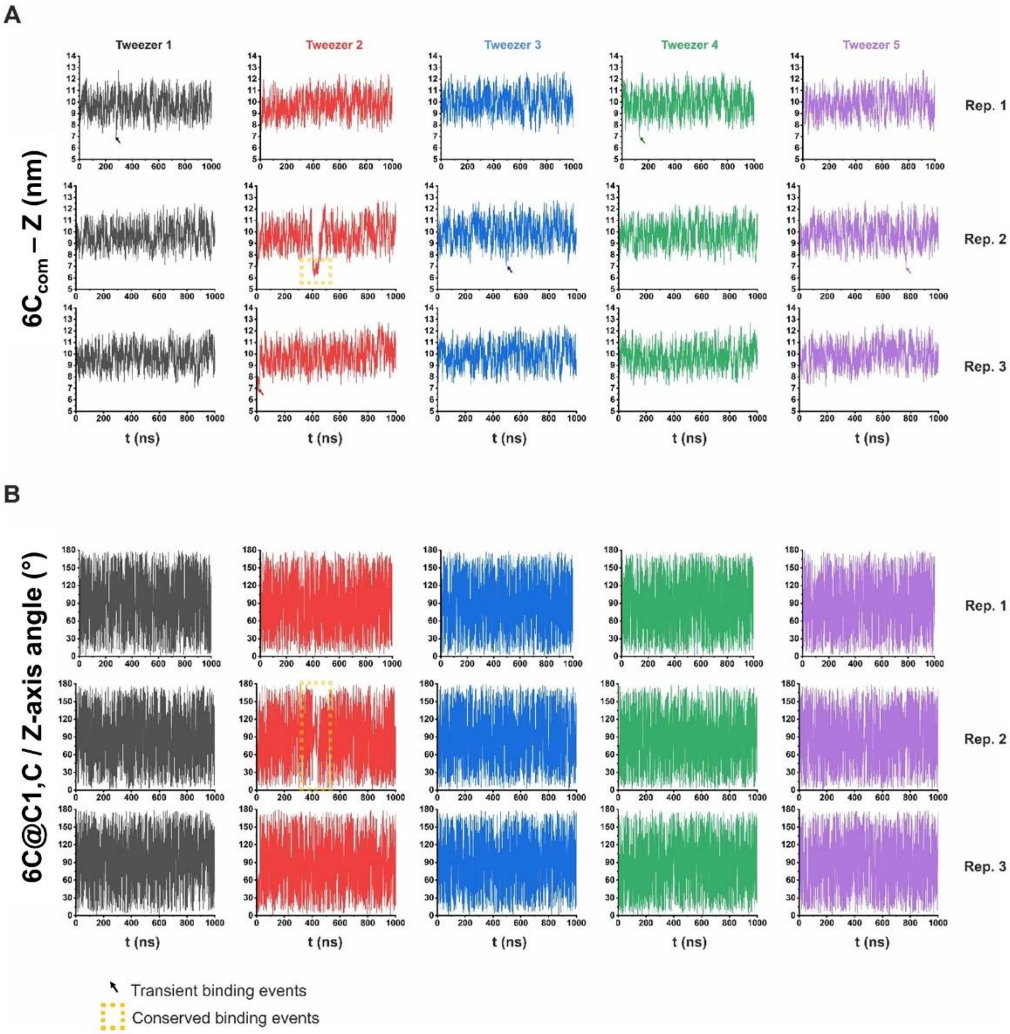

Figure S7. A) Binding events of 6C with the upper leaflet of POPG membrane during the MD simulations. The arrows mark transient binding events while the yellow dotted boxes indicate conserved binding events. Each row shows the results per replica (Rep. 1, Rep. 2, Rep. 3) for each tweezer molecule (columns). The Y-axes correspond to the Z-coordinate of the center of mass of the tweezer.
B) Tweezer orientation with respect to the membrane. Time evolution of the angle μ (Fig. S5) during the simulation. The yellow dotted box indicates the conserved binding event.

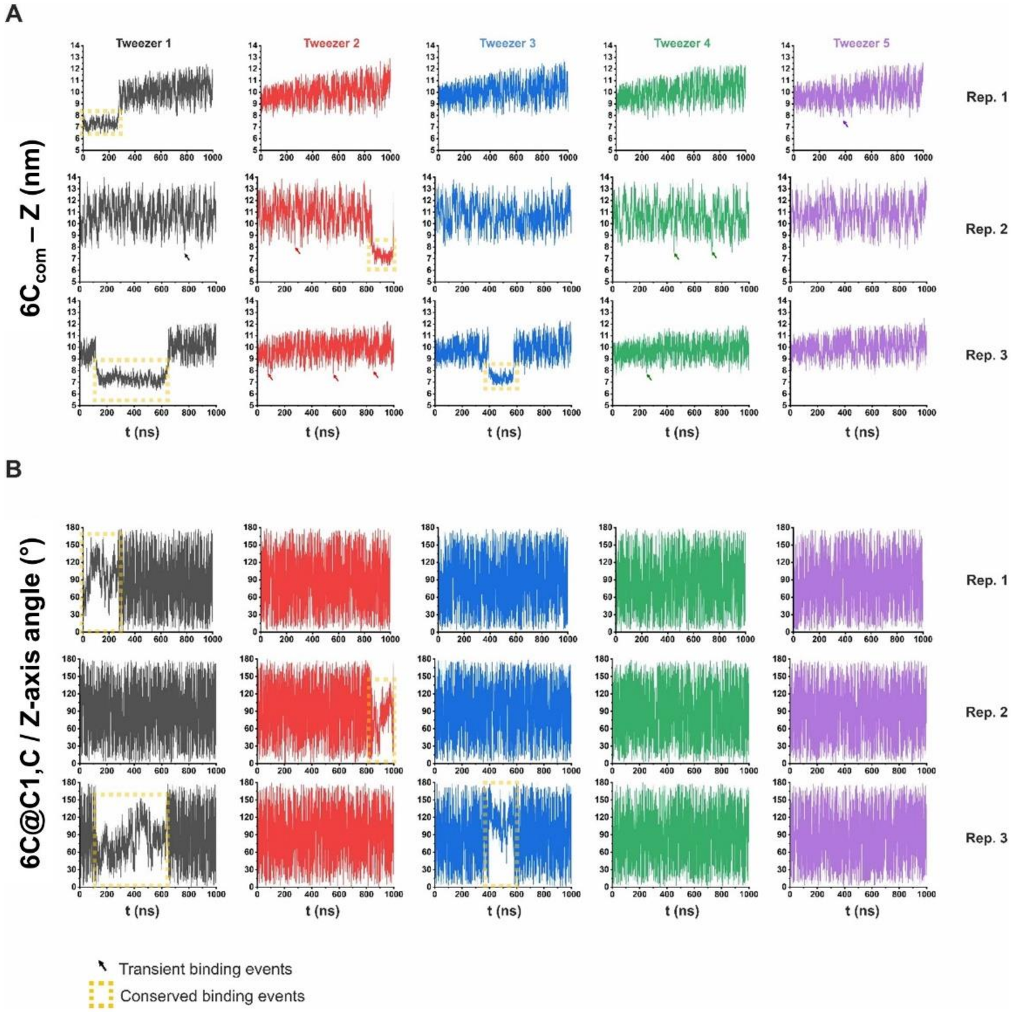

Fig. S8. A) Binding events of 6C with the upper leaflet of POPA membrane during the MD simulations. The arrows mark transient binding events while the yellow dotted boxes indicate conserved binding events. Each row shows the results per replica (Rep. 1, Rep. 2, or Rep. 3) for each tweezer molecule (columns). The Y-axes correspond to the Z-coordinate of the center of mass of the tweezer.

B) Tweezer orientation with respect to the membrane. Time evolution of the angle μ (Fig. S5) during the simulation. The yellow dotted box indicates the conserved binding event.

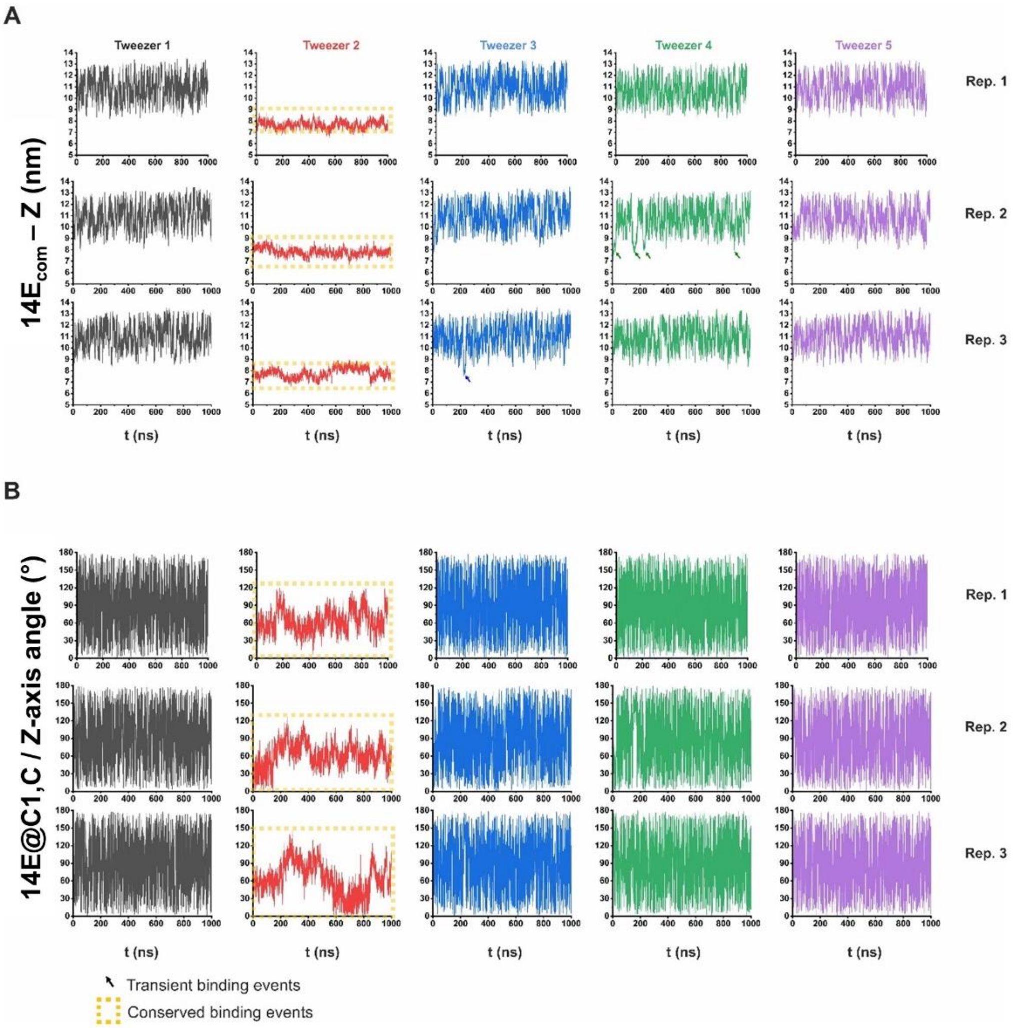

Fig. S9. A) Binding events of 14E with the upper leaflet of POPA membrane during the MD simulations. The arrows mark transient binding events while the yellow dotted boxes indicate conserved binding events. Each row shows the results per replica (Rep. 1, Rep. 2, or Rep. 3) for each tweezer molecule (columns). The Y-axes correspond to the Z-coordinate of the center of mass of the tweezer.
B) Tweezer orientation with respect to the membrane. Time evolution of the angle µ (Fig. S5) during the simulation. The yellow dotted box indicates the conserved binding event.

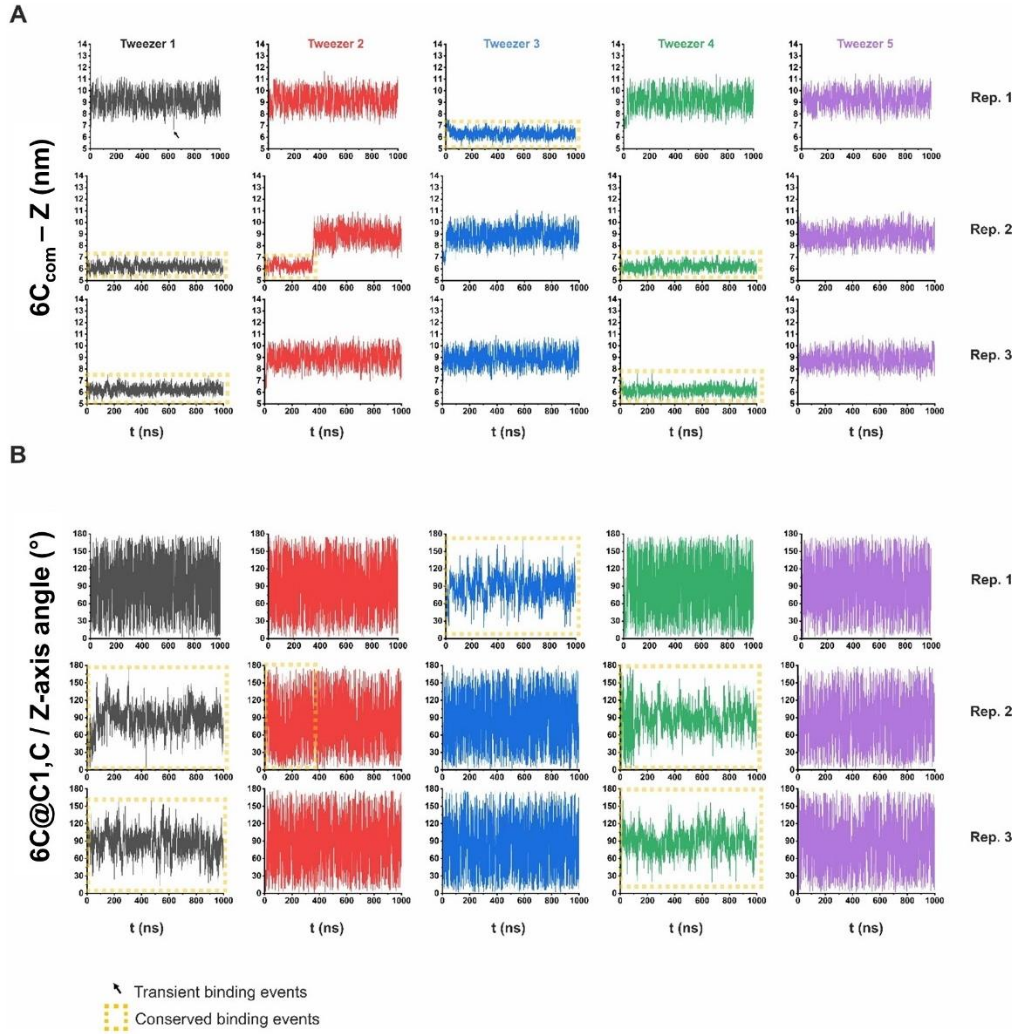

Fig. S10. A) Binding events of 6C with the upper leaflet of POPS membrane during the MD simulations. The arrow marks the transient binding event while the yellow dotted boxes indicate conserved binding events. Each row shows the results per replica (Rep. 1, Rep. 2, or Rep. 3) for each tweezer molecule (columns). The Y-axes correspond to the Z-coordinate of the center of mass of the tweezer.

B) Tweezer orientation with respect to the membrane. Time evolution of the angle μ (Fig. S5) during the simulation. The yellow dotted box indicates the conserved binding event.

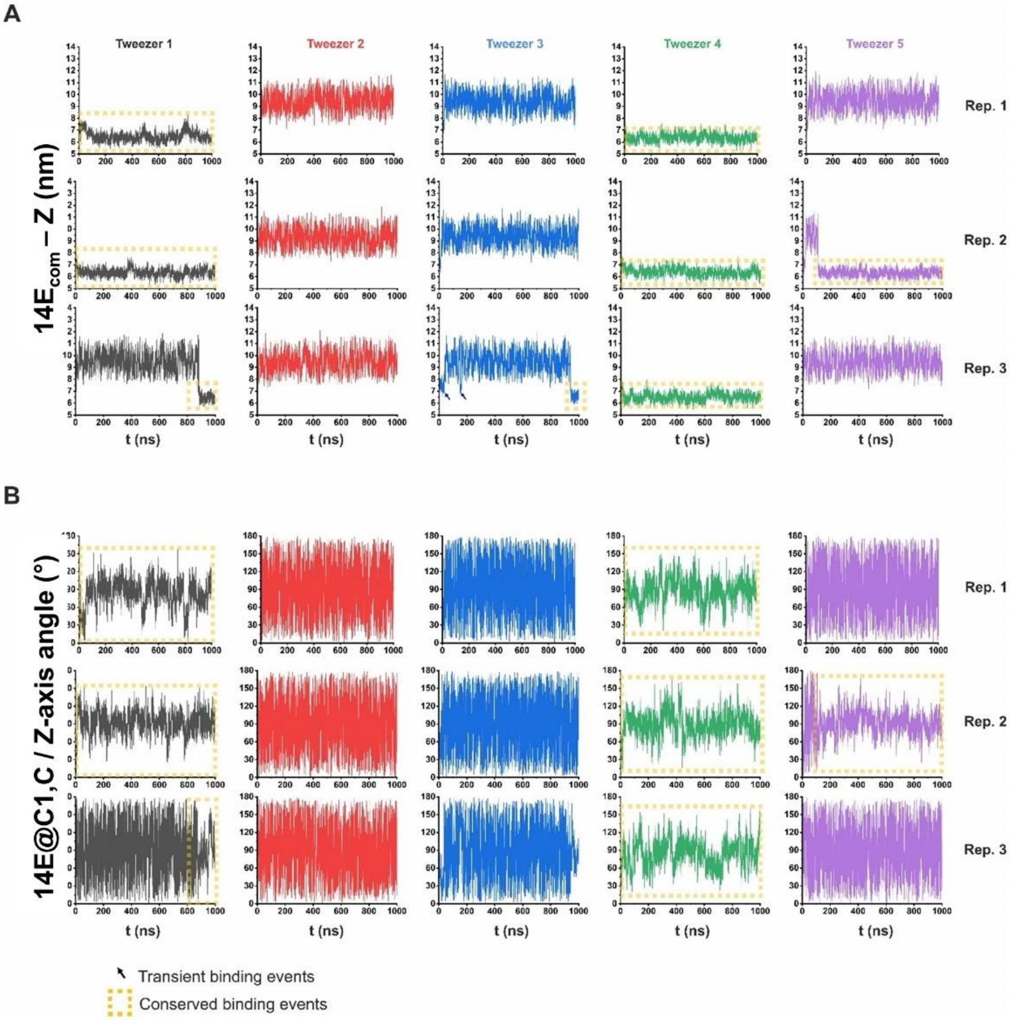

Fig. S11. A) Binding events of 14E with the POPS membrane during the MD simulations. The arrow marks the transient binding event while the yellow dotted boxes indicate conserved binding events. Each row shows the results per replica (Rep. 1, Rep. 2, or Rep. 3) for each tweezer molecule (columns). The Y-axes correspond to the Z-coordinate of the center of mass of the tweezer.

B) Tweezer orientation with respect to the membrane. Time evolution of the angle μ (Fig. S5) during the simulation. The yellow dotted box indicates the conserved binding event.

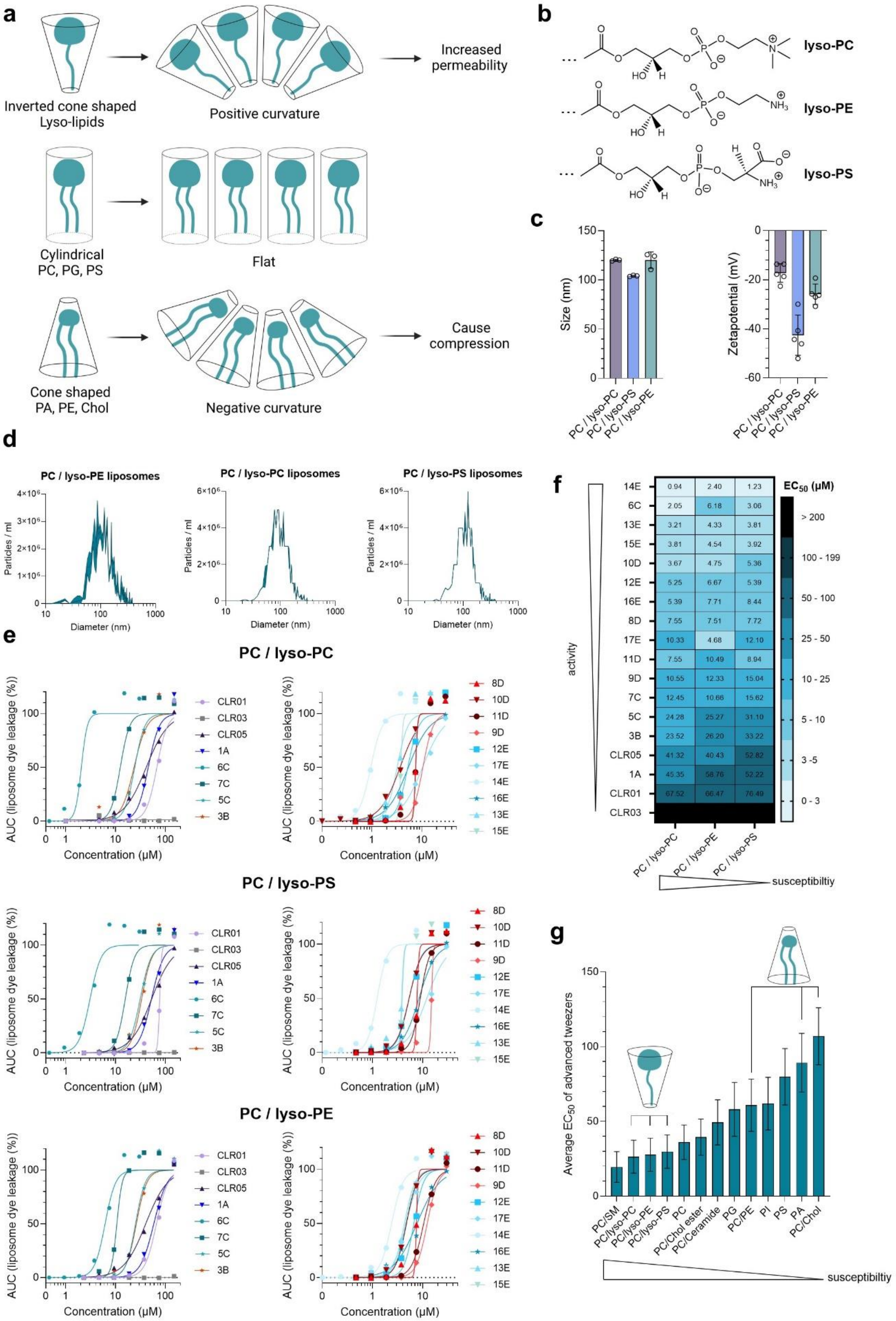
a
Inverted cone shaped
Lyso-lipids
Positive curvature
Increased
permeability
Cylindrical
PC, PG, PS
Flat
Cone shaped
PA, PE, Chol
Negative curvature
Cause
compression
b
lyso-PC
lyso-PE
lyso-PS
c
Size (nm)
Zetapotential (mV)
PC / lyso-PC
PC / lyso-PS
PC / lyso-PE
d
PC / lyso-PE liposomes
PC / lyso-PC liposomes
PC / lyso-PS liposomes
Particles / ml
Diameter (nm)
e
PC / lyso-PC
PC / lyso-PS
PC / lyso-PE
AUC (liposome dye leakage (%))
Concentration (µM)
CLR01
CLR03
CLR05
1A
6C
7C
5C
3B
8D
10D
11D
9D
12E
17E
14E
16E
13E
15E
f
activity
EC50 (µM)
14E 0.94 2.40 1.23
6C 2.05 6.18 3.06
13E 3.21 4.33 3.81
15E 3.81 4.54 3.92
10D 3.67 4.75 5.36
12E 5.25 6.67 5.39
16E 5.39 7.71 8.44
8D 7.55 7.51 7.72
17E 10.33 4.68 12.10
11D 7.55 10.49 8.94
9D 10.55 12.33 15.04
7C 12.45 10.66 15.62
5C 24.28 25.27 31.10
3B 23.52 26.20 33.22
CLR05 41.32 40.43 52.82
1A 45.35 58.76 52.22
CLR01 67.52 66.47 76.49
CLR03
> 200
100 - 199
50 - 100
25 - 50
10 - 25
5 - 10
3 -5
0 - 3
PC / lyso-PC
PC / lyso-PE
PC / lyso-PS
susceptibiltiy
g
Average EC50 of advanced tweezers
PC/SM
PC/lyso-PC
PC/lyso-PE
PC/lyso-PS
PC
PC/Chol ester
PC/Ceramide
PG
PC/PE
PI
PS
PA
PC/Chol
susceptibiltiy

Figure S12. Tweezer activity is increased in presence of lyso-lipid liposomes. a, Overview of lipid shapes. Created in BioRender. Lawrenz, J. (2025) https://BioRender.com/5ha6nay. Adapted from Peeters and Piëel al. 2022[24] (Department of Cell Biology, Erasmus MC, Rotterdam, The Netherlands; CC BY 4.0; Copyright© The Authors, https://doi.org/10.3390/cells11030469). **b,** Chemical structure of lyso-lipid head groups. Created with ChemDraw. **c,** Particle size and zeta potential of 100 nm sized mixed (95/5 mol%) liposomes, filled with 50 mM carboxyfluorescein were analysed by NTA. Measurement was conducted in 3 (NTA) to 5 (zeta potential) acquisitions and shown as mean values ± SD. **d**, Raw data of particle size and number from c. **e,** Liposomes (2.5 x 10^{10} particles/ml) were incubated with increasing concentrations of tweezers for 30 min and fluorescence was recorded every minute at 485 nm excitation and 528 nm emission. Baseline was measured for 5 min in absence of tweezers and maximum fluorescence was recorded after the addition of 1 % Triton X-100. Values were corrected for baseline and normalized to maximum fluorescence. Graphs show area under the curve in means of one experiment performed in triplicates. **f,** Overview tweezer activity against various liposome types derived from e. **g,** Average EC_{50} values derived from f and Fig. 2c blotted according to the liposome susceptibility to tweezers. Modified from Weil, 2023[23] (CC BY 4.0; https://creativecommons.org/licenses/by/4.0/).

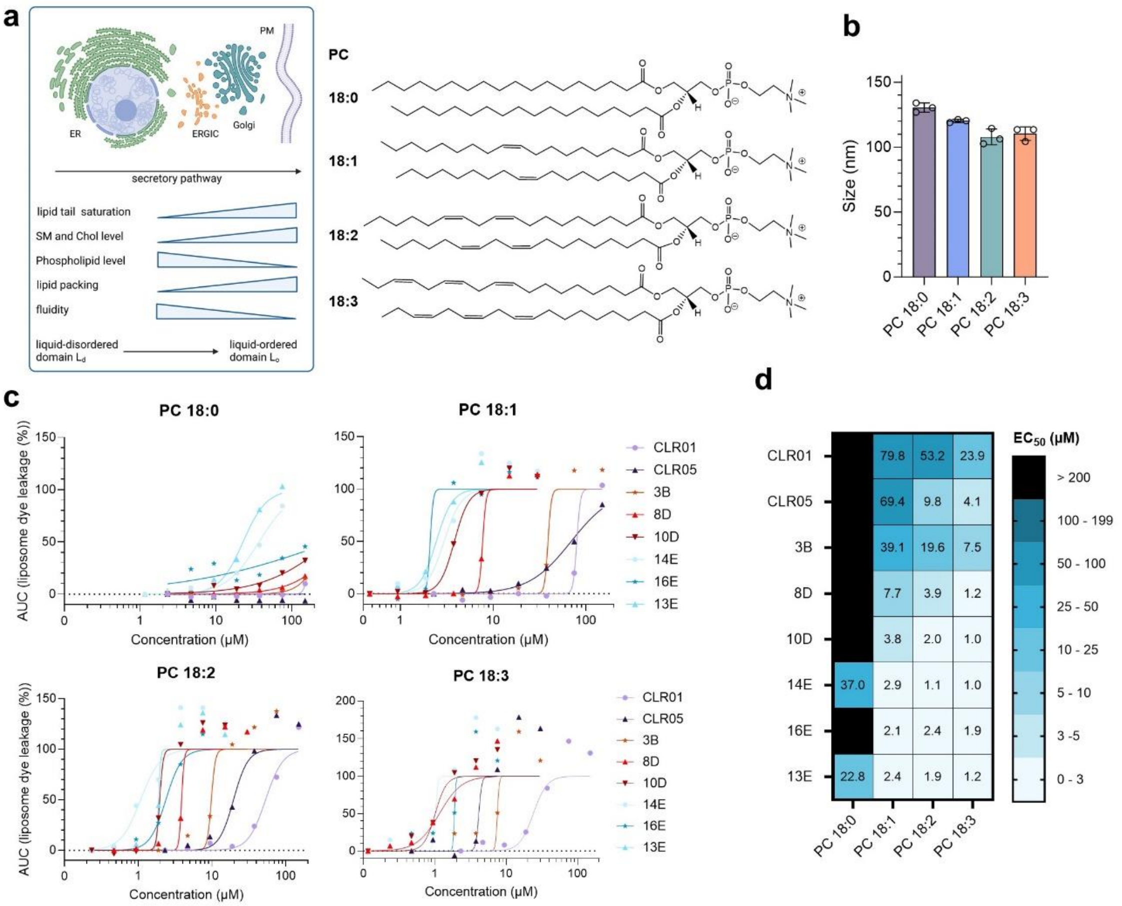

Fig. S13. Membranolytic activity of tweezers is influenced by the saturation degree of lipids. a, Characteristics of the cellular membranes along the secretory pathway and chemical structures of the lipid PC with different saturation degrees. Created in BioRender. Lawrenz, J. (2025) https://BioRender.com/5ha6nay and ChemDraw. **b,** Particle size of 100 nm sized uniform liposomes, filled with 50 mM carboxyfluorescein were analysed by NTA. Measurement was conducted in 3 acquisitions and shown as mean values ± SD. **c,** Liposomes (2.5 x 10^{10} particles/ml) were incubated with increasing concentrations of tweezers for 30 min and fluorescence was recorded every minute at 485 nm excitation and 528 nm emission. Baseline was measured for 5 min in absence of tweezers and maximum fluorescence was recorded after the addition of 1 % Triton X-100. Values were corrected for baseline and normalized to maximum fluorescence. Graphs show area under the curve in means of one experiment performed in triplicates. **d,** Overview tweezer activity against various liposome types derived from c.

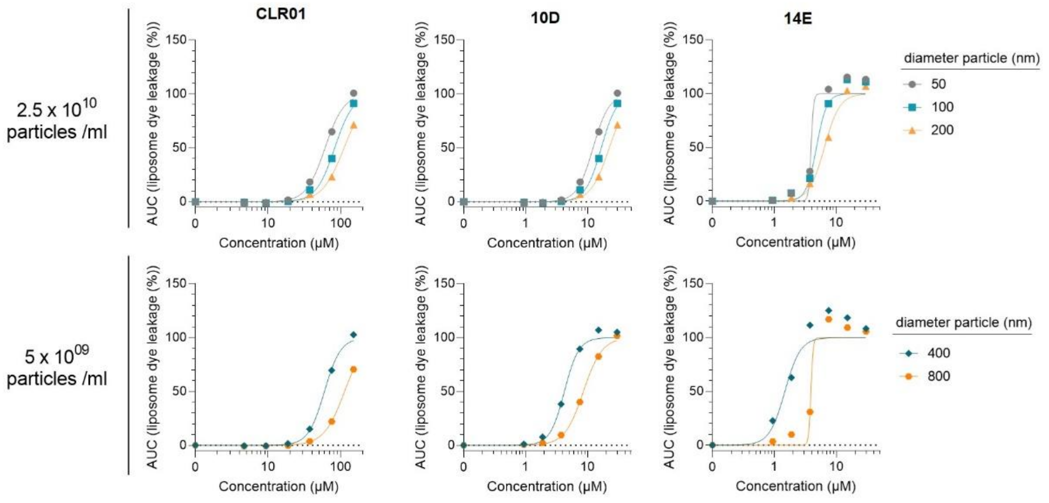

Fig. S14. Tweezers disrupt smaller virus-like liposomes more efficiently. Defined number of virus-like liposomes (DOPC/SM/Chol (45/25/30 mol%)) filled with 50 mM carboxyfluorescein and sized with a membrane ranging from 50 to 800 nm in pore diameter were incubated with serial dilution of tweezers for 30 min and dye leakage was recorded every minute at 485 nm excitation and 528 nm emission. Baseline was measured for 5 min in absence of tweezers and maximum fluorescence was recorded after the addition of 1 % Triton X-100. Values were corrected for baseline and normalized to maximum fluorescence. Graphs show area under the curve for each tweezer concentration from one experiment performed in triplicates. Modified from Weil, 2023[23] (CC BY 4.0; https://creativecommons.org/licenses/by/4.0/).

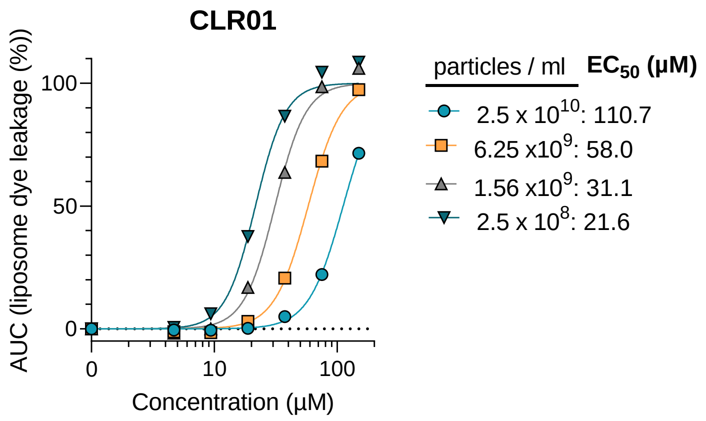

Fig. S15. Tweezer activity correlate inversely with number of liposomes. Escalating concentrations of 200 nm sized virus-like liposomes (DOPC/SM/Chol (45/25/30 mol%)) filled with 50 mM carboxyfluorescein were incubated with serial dilution of tweezers for 30 min and dye leakage was recorded every minute at 485 nm excitation and 528 nm emission. Baseline was measured for 5 min in absence of tweezers and maximum fluorescence was recorded after the addition of 1 % Triton X-100. Values were corrected for baseline and normalized to maximum fluorescence. Graphs show area under the curve for each tweezer concentration from one experiment performed in triplicates. Modified from Weil, 2023[23] (CC BY 4.0; https://creativecommons.org/licenses/by/4.0/).

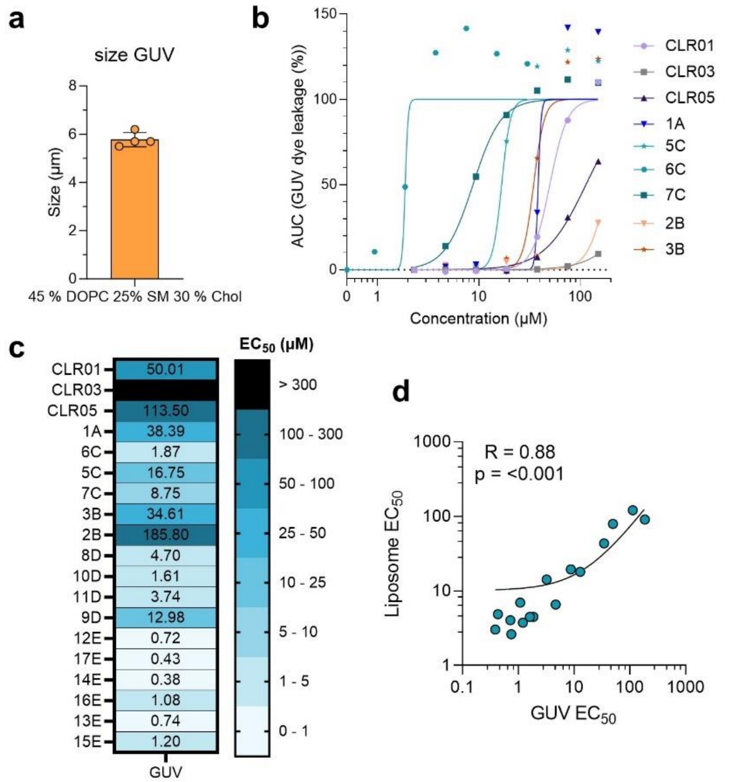

Fig. S16. Characterization of giant unilamellar vesicles (GUV´s). a, GUV´s consisting of DOPC/SM/Chol (45/25/30 mol%) are filled with 50 mM carboxyfluorescein and were produced by the PAPYRUS method[17]. Shown are mean values of GUV diameter ± SD in quadruplicates. **b,** GUVs, at a concentration of 1.0 x 10^4 particles/ml were treated with serial dilutions of tweezers for 30 minutes, dye leakage was monitored at 485 nm excitation and 528 nm emission. Baselines were established without tweezers for 5 minutes, and maximum fluorescence was determined after adding 1% Triton X-100. The resulting graphs plot the area under the curve for each tweezer concentration, corrected for baseline and normalized to the maximum fluorescence, based on a single experiment performed in triplicate. **c,** Overview tweezer activity against GUV´s derived from b. **d,** Correlation analysis of EC_{50} values derived from c and previous published data[1]. Analysis was assessed by Spearman Correlation, two-tailed p value. Modified from Weil, 2023[23] (CC BY 4.0; https://creativecommons.org/licenses/by/4.0/).

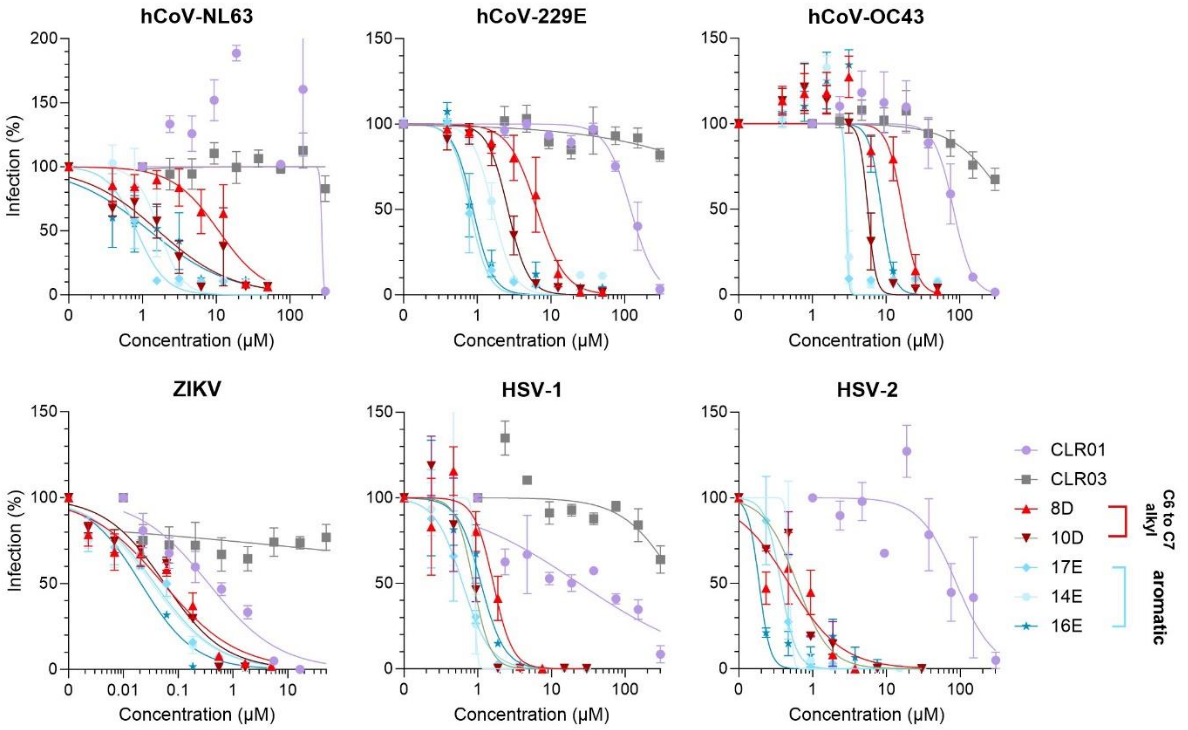

Fig. S17. Broad-spectrum antiviral activity of advanced tweezers 1. a, To assess antiviral activity of tweezers, HCoV-NL63 (MOI 0.01), -229E (MOI 0.002) or -OC43 (MOI 0.006) were incubated with tweezers for 30 min at 33°C before inoculation onto Caco2 (in case of hCoV-NL63) or Huh-7 cells. Two (hCoV-229E), three (hCoV-OC43) or six (hCoV-NL63) days post infection, viral nucleocapsid was quantified by in-cell ELISA. ZIKV (MOI 0.15) was incubated with titrated tweezers for 30 min at 37 °C before inoculation of Vero E6 cells. Infection rates were accessed two days later by in-cell Elisa detecting flavivirus protein E. HSV-1 (MOI 0.05) and HSV-2 (MOI 0.05) encoding GFP were incubated with titrated tweezers for 15 min at 37°C and used for inoculation onto ELVIS reporter cells. Two day later, infection rates were determined by quantifying β-galactosidase activity in cellular lysates. Graphs represent mean values ± SEM normalized to mock infected controls and are derived from two (in case of HSV-1 and HSV-2) or three independent experiments each performed in triplicates. Modified from Weil, 2023[23] (CC BY 4.0; https://creativecommons.org/licenses/by/4.0/).

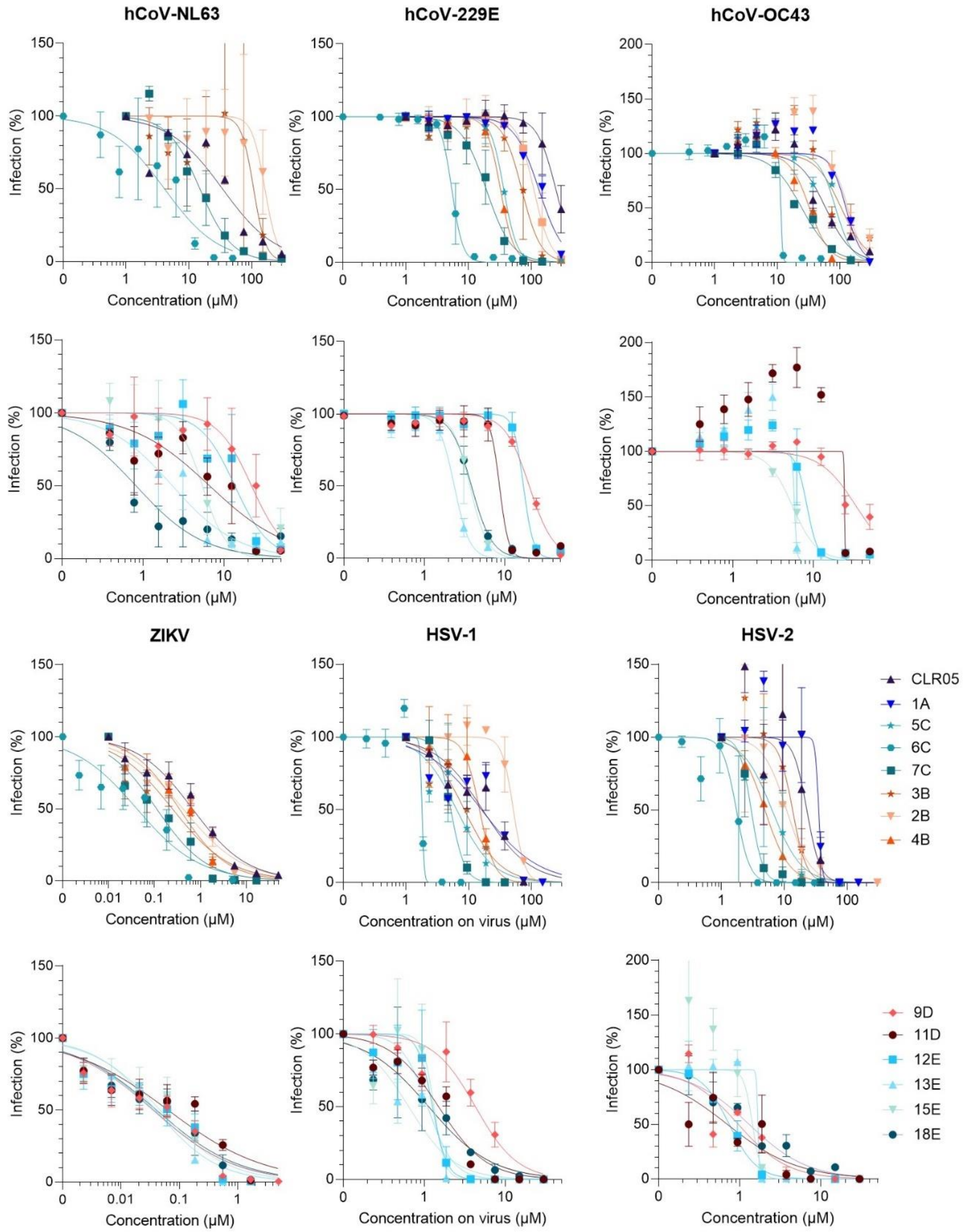

Fig. S18. Broad-spectrum antiviral activity of advanced tweezers 2. a, To assess antiviral activity of tweezers, HCoV-NL63 (MOI 0.01), -229E (MOI 0.002) or -OC43 (MOI 0.006) were incubated with tweezers for 30 min at 33°C before inoculation onto Caco2 (in case of hCoV-NL63) or Huh-7 cells. Two (hCoV-229E), three (hCoV-OC43) or six (hCoV-NL63) days post infection, viral nucleocapsid was quantified by in-cell ELISA. ZIKV (MOI 0.15) was incubated with titrated tweezers for 30 min at 37 °C before inoculation of Vero E6 cells. Infection rates were accessed two days later by in-cell Elisa detecting flavivirus protein E. HSV-1 (MOI 0.05) and HSV-2 (MOI 0.05) encoding GFP were incubated with titrated tweezers for 15 min at 37°C and used for inoculation onto ELVIS reporter cells. Two day later, infection rates were determined by quantifying β-galactosidase activity in cellular lysates.

Graphs represent mean values ± SEM normalized to mock infected controls and are derived from two (in case of HSV-1 and HSV-2) or three independent experiments each performed in triplicates. Modified from Weil, 2023[23] (CC BY 4.0; https://creativecommons.org/licenses/by/4.0/).

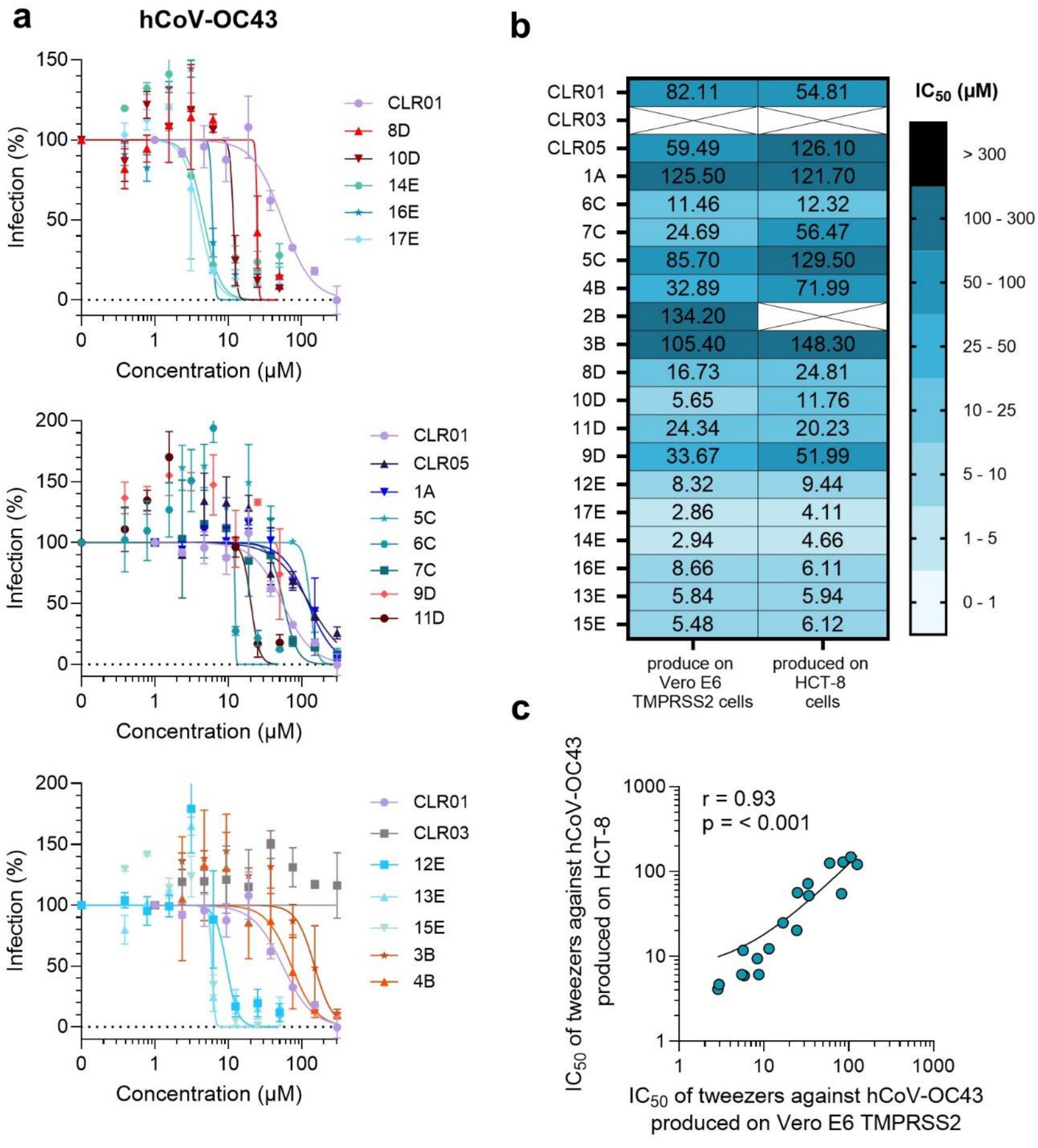

Figure S19. Propagation of hCoV-OC43 in two different cells lines does not impair tweezer activity. a, Titrated tweezers were incubated with hCoV-OC43 (MOI 0.006) produced on HCT-8 cells for 30 min at 33°C before inoculation onto Huh-7 cells. Three days post infection, viral nucleocapsid was quantified by in-cell ELISA. Graphs show mean values ± SEM of two independent experiments conducted in triplicates. **b,** IC_{50} of tweezers against hCoV-OC43 propagated on TMPRSS2 expressing Vero E6 (derived from Fig. 6b) or on HCT-8 cells (derived from a). **c,** Correlation analysis of tweezer activity against hCoV-OC43 produced on two different cell lines. IC_{50} values from were analysed by Spearman Correlation, two-tailed p value. Modified from Weil, 2023[23] (CC BY 4.0; https://creativecommons.org/licenses/by/4.0/).

Supplementary References

(1) Weil, T.; Kirupakaran, A.; Le, M.-H.; Rebmann, P.; Mieres-Perez, J.; Issmail, L.; Conzelmann, C.; Müller, J. A.; Rauch, L.; Gilg, A.; Wettstein, L.; Groß, R.; Read, C.; Bergner, T.; Pålsson, S. A.; Uhlig, N.; Eberlein, V.; Wöll, H.; Klärner, F.-G.; Stenger, S.; Kümmerer, B. M.; Streeck, H.; Fois, G.; Frick, M.; Braubach, P.; Spetz, A.-L.; Grunwald, T.; Shorter, J.; Sanchez-Garcia, E.; Schrader, T.; Münch, J. Advanced Molecular Tweezers with Lipid Anchors against SARS-CoV-2 and Other Respiratory Viruses. *JACS Au* **2022**, *2* (9), 2187–2202. https://doi.org/10.1021/jacsau.2c00220.

(2) Driggers, R. W.; Ho, C.-Y.; Korhonen, E. M.; Kuivanen, S.; Jääskeläinen, A. J.; Smura, T.; Rosenberg, A.; Hill, D. A.; DeBiasi, R. L.; Vezina, G.; Timofeev, J.; Rodriguez, F. J.; Levanov, L.; Razak, J.; Iyengar, P.; Hennenfent, A.; Kennedy, R.; Lanciotti, R.; du Plessis, A.; Vapalahti, O. Zika Virus Infection with Prolonged Maternal Viremia and Fetal Brain Abnormalities. *New England Journal of Medicine* **2016**, *374* (22), 2142–2151. https://doi.org/10.1056/nejmoa1601824.

(3) Weil, T.; Lawrenz, J.; Seidel, A.; Münch, J.; Müller, J. A. Immunodetection Assays for the Quantification of Seasonal Common Cold Coronaviruses OC43, NL63, or 229E Infection Confirm Nirmatrelvir as Broad Coronavirus Inhibitor. *Antiviral Research* **2022**, *203*, 105343. https://doi.org/10.1016/j.antiviral.2022.105343.

(4) Aubry, M.; Richard, V.; Green, J.; Broult, J.; Musso, D. Inactivation of Zika Virus in Plasma with Amotosalen and Ultraviolet A Illumination. *Transfusion* **2016**, *56* (1), 33–40. https://doi.org/10.1111/trf.13271.

(5) Proffitt, M. R.; Schindler, S. A. Rapid Detection of HSV with an Enzyme-Linked Virus Inducible System™ (ELVIS™) Employing a Genetically Modified Cell Line. *Clinical and Diagnostic Virology* **1995**, *4* (2), 175–182. https://doi.org/10.1016/0928-0197(95)00011-V.

(6) Wu, E. L.; Cheng, X.; Jo, S.; Rui, H.; Song, K. C.; Dávila-Contreras, E. M.; Qi, Y.; Lee, J.; Monje-Galvan, V.; Venable, R. M.; Klauda, J. B.; Im, W. CHARMM-GUI Membrane Builder toward Realistic Biological Membrane Simulations. *Journal of Computational Chemistry* **2014**, *35* (27), 1997–2004. https://doi.org/10.1002/jcc.23702.

(7) Jo, S.; Kim, T.; Iyer, V. G.; Im, W. CHARMM-GUI: A Web-Based Graphical User Interface for CHARMM. *Journal of Computational Chemistry* **2008**, *29* (11), 1859–1865. https://doi.org/10.1002/jcc.20945.

(8) Ruiz-Blanco, Y. B.; Sanchez-Garcia, E. CL-FEP: An End-State Free Energy Perturbation Approach. *J. Chem. Theory Comput.* **2020**, *16* (3), 1396–1410. https://doi.org/10.1021/acs.jctc.9b00725.

(9) Malishev, R.; Salinas, N.; Gibson, J.; Eden, A. B.; Mieres-Perez, J.; Ruiz-Blanco, Y. B.; Malka, O.; Kolusheva, S.; Klärner, F.-G.; Schrader, T.; Sanchez-Garcia, E.; Wang, C.; Landau, M.; Bitan, G.; Jelinek, R. Inhibition of Staphylococcus Aureus Biofilm-Forming Functional Amyloid by Molecular Tweezers. *Cell Chem Biol* **2021**, *28* (9), 1310-1320.e5. https://doi.org/10.1016/j.chembiol.2021.03.013.

(10) Jorgensen, W. L.; Chandrasekhar, J.; Madura, J. D.; Impey, R. W.; Klein, M. L. Comparison of Simple Potential Functions for Simulating Liquid Water. *The Journal of Chemical Physics* **1983**, *79* (2), 926–935. https://doi.org/10.1063/1.445869.

(11) Abraham, M. J.; Murtola, T.; Schulz, R.; Páll, S.; Smith, J. C.; Hess, B.; Lindahl, E. GROMACS: High Performance Molecular Simulations through Multi-Level Parallelism from Laptops to Supercomputers. *SoftwareX* **2015**, *1–2*, 19–25. https://doi.org/10.1016/j.softx.2015.06.001.

(12) Huang, J.; Rauscher, S.; Nawrocki, G.; Ran, T.; Feig, M.; de Groot, B. L.; Grubmüller, H.; MacKerell, A. D. CHARMM36m: An Improved Force Field for Folded and

Intrinsically Disordered Proteins. *Nat Methods* **2017**, *14* (1), 71–73. https://doi.org/10.1038/nmeth.4067.
(13) Darden, T.; York, D.; Pedersen, L. Particle Mesh Ewald: An N·log(N) Method for Ewald Sums in Large Systems. *The Journal of Chemical Physics* **1993**, *98* (12), 10089–10092. https://doi.org/10.1063/1.464397.
(14) Izaguirre, J. A.; Catarello, D. P.; Wozniak, J. M.; Skeel, R. D. Langevin Stabilization of Molecular Dynamics. *The Journal of Chemical Physics* **2001**, *114* (5), 2090–2098. https://doi.org/10.1063/1.1332996.
(15) Martyna, G. J.; Tobias, D. J.; Klein, M. L. Constant Pressure Molecular Dynamics Algorithms. *The Journal of Chemical Physics* **1994**, *101* (5), 4177–4189. https://doi.org/10.1063/1.467468.
(16) Weil, T.; Groß, R.; Röcker, A.; Bravo-Rodriguez, K.; Heid, C.; Sowislok, A.; Le, M.-H.; Erwin, N.; Dwivedi, M.; Bart, S.; Bates, P.; Wettstein, L.; Müller, J.; Harms, M.; Sparrer, K.; Ruiz-Blanco, Y. B.; Stuerzel, C.; von Einem, J.; Lippold, S.; Read, C.; Walther, P.; Hebel, M.; Kreppel, F.; Klärner, F.-G.; Bitan, G.; Ehrmann, M.; Weil, T.; Winter, R.; Schrader, T.; Shorter, J.; Sanchez-Garcia, E.; Muench, J. Supramolecular Mechanism of Viral Envelope Disruption by Molecular Tweezers. *Journal of the American Chemical Society* **2020**, jacs.0c06400. https://doi.org/10.1021/jacs.0c06400.
(17) Kresse, K. M.; Xu, M.; Pazzi, J.; García-Ojeda, M.; Subramaniam, A. B. Novel Application of Cellulose Paper As a Platform for the Macromolecular Self-Assembly of Biomimetic Giant Liposomes. *ACS Applied Materials and Interfaces* **2016**, *8* (47), 32102–32107. https://doi.org/10.1021/acsami.6b11960.
(18) R Core Team. *R: A language and environment for statistical computing. R Foundation for Statistical Computing*. https://www.r-project.org/ (accessed 2023-03-17).
(19) Josse, J.; Husson, F. missMDA: A Package for Handling Missing Values in Multivariate Data Analysis. *Journal of Statistical Software* **2016**, *70*, 1–31. https://doi.org/10.18637/jss.v070.i01.
(20) Lê, S.; Josse, J.; Husson, F. FactoMineR: An R Package for Multivariate Analysis. *Journal of Statistical Software* **2008**, *25*, 1–18. https://doi.org/10.18637/jss.v025.i01.
(21) Kassambara, A.; Mundt, F. Factoextra: Extract and Visualize the Results of Multivariate Data Analyses, 2020. https://CRAN.R-project.org/package=factoextra (accessed 2023-03-17).
(22) Wickham, H.; Averick, M.; Bryan, J.; Chang, W.; McGowan, L. D.; François, R.; Grolemund, G.; Hayes, A.; Henry, L.; Hester, J.; Kuhn, M.; Pedersen, T. L.; Miller, E.; Bache, S. M.; Müller, K.; Ooms, J.; Robinson, D.; Seidel, D. P.; Spinu, V.; Takahashi, K.; Vaughan, D.; Wilke, C.; Woo, K.; Yutani, H. Welcome to the Tidyverse. *Journal of Open Source Software* **2019**, *4* (43), 1686. https://doi.org/10.21105/joss.01686.
(23) Weil, T. C. Advanced Molecular Tweezers as Broad-Spectrum Antivirals. Dissertation, Universität Ulm, 2023. https://doi.org/10.18725/OPARU-51003.
(24) Peeters, B. W. A.; Piët, A. C. A.; Fornerod, M. Generating Membrane Curvature at the Nuclear Pore: A Lipid Point of View. *Cells* **2022**, *11* (3), 469. https://doi.org/10.3390/cells11030469.
